# Protein arginine methylation regulates anti-inflammatory programming in response to aging stress

**DOI:** 10.1016/j.isci.2025.113095

**Published:** 2025-07-10

**Authors:** Yao Yuan, Kaori Motomura, Jun-Dal Kim, Kowit Hengphasatporn, Koichiro Kako, Syunsuke Maruhashi, Fumiya Kasai, Hayase Mizukami, Sachiko Toma-Fukai, Masafumi Muratani, Yasuteru Shigeta, Hiroaki Daitoku, Akiyoshi Fukamizu

**Affiliations:** 1 Life Science Center for Survival Dynamics, Tsukuba Advanced Research Alliance (TARA), University of Tsukuba, Ibaraki 305-8575, Japan; 2 Human Biology, School of Integrative and Global Majors, University of Tsukuba, Tsukuba Ibaraki 305-8575, Japan; 3 AMED-CREST, Japan Agency for Medical Research and Development, Chiyoda-ku, Tokyo, Japan; 4 Division of Complex Bioscience Research, Department of Research and Development, Institute of National Medicine, University of Toyama, Toyama, Japan; 5 Center for Computational Sciences, University of Tsukuba, Ibaraki 305-8577, Japan; 6 Faculty of Life and Environmental Sciences, University of Tsukuba, Ibaraki 305-8575, Japan; 7 Agro-Bioresources Science and Technology, University of Tsukuba, 1-1-1 Tennodai, Tsukuba, Ibaraki 305-8572, Japan; 8 Life and Agricultural Sciences, University of Tsukuba, 1-1-1 Tennodai, Tsukuba, Ibaraki 305-8572, Japan; 9 Graduate School of Science and Technology, Nara Institute of Science and Technology, Ikoma, Nara 630-0192, Japan; 10 Department of Genome Biology, Institute of Medicine, University of Tsukuba, Ibaraki 305-8575, Japan; 11 Tsukuba Institute for Advanced Research (TIAR), University of Tsukuba, 1-1-1 Tennodai, Tsukuba, Ibaraki 305-8577, Japan

**Keywords:** Epigenetics, mmunology, ranscriptomics

## Abstract

PRMT1 is a key enzyme responsible for protein arginine methylation, which regulates various cellular processes. However, its physiological significance at whole-body level remains unknown due to embryonic lethality of Prmt1-null mice. Despite only one amino acid difference at position 179, molecular dynamics simulations and biochemical assays showed that human PRMT1 exhibits enhanced methyltransferase activity compared to its mouse counterpart. Capitalizing on this finding, we generated humanized PRMT1 knock-in mice (huMice) carrying the H179Y substitution. Notably, huMice displayed distinct transcriptomic signatures associated with attenuated inflammatory responses compared to wild-type mice in an age-dependent manner. In particular, huMice exhibited reduced pro-inflammatory cytokines production following lipopolysaccharide (LPS) challenge at 12 months, revealing that heightened PRMT1 activity confers a physiological advantage in alleviating age-related inflammatory stress. Our findings underscore PRMT1’s key role in the modulation of anti-inflammatory programming at the organismal level and open avenues for leveraging this knowledge in developing mitigation strategies against inflammaging.

## Highlights

• Human PRMT1 (hPRMT1) shows higher enzymatic activity than mouse PRMT1 (mPRMT1)
• A single amino acid difference underlies the catalytic advantage of hPRMT1
• Humanized PRMT1 mice (huMice) show transcriptomic signatures of stress resistance
• Old huMice exhibit increased resilience to LPS-induced systemic inflammation

## Graphical abstract

**Figure:**
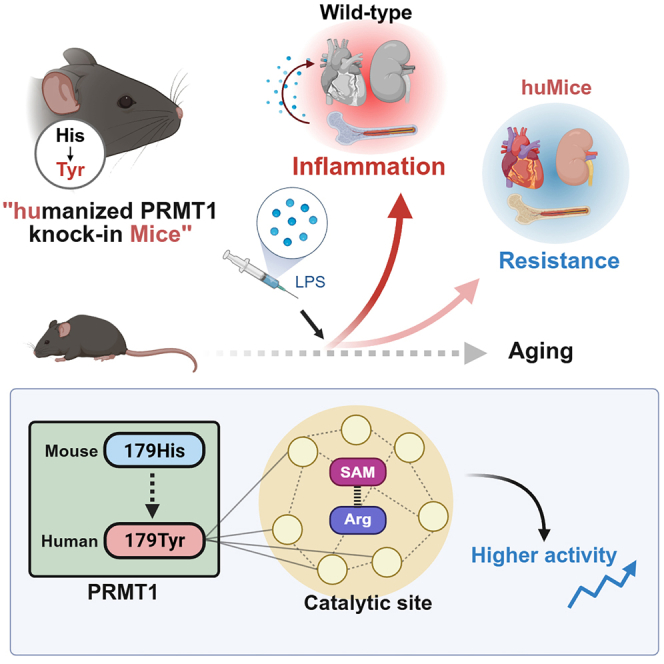

## Teaser

Epigenetics; Immunology; Transcriptomics

## Introduction

Protein arginine methylation has attracted growing attention as a key post-translational modification (PTM) found in a variety of cellular proteins.^1^ In mammals, nine protein arginine methyltransferases (PRMTs) are identified, and they catalyze the transfer of a methyl group using *S*-adenosylmethionine (SAM) as the methyl donor to the guanidino nitrogen atoms of arginine residues in proteins. PRMTs have been divided into three types based on their reaction products: type I PRMTs; PRMT1, PRMT2, PRMT3, CARM1 (also known as PRMT4), PRMT6, and PRMT8 catalyze the formation of mono-methylarginine (MMA) and asymmetric dimethylarginine (ADMA). In contrast, type II PRMTs, including PRMT5 and PRMT9, generate MMA and symmetric dimethylarginine (SDMA). For the sole Type III enzyme, PRMT7 produces MMA, but neither ADMA nor SDMA. We and other groups have reported that the three types of PRMT activity are evolutionary conserved from nematode to humans.^2^

Recent methodological developments provide routes for investigating global protein arginine methylation in biological samples. For example, an improved method with high-resolution mass spectrometry identified 8030 arginine methylation sites within 3300 human proteins in HEK293 cells, indicating that this occurrence is equivalent to phosphorylation and ubiquitination.^3^ Another strategy utilizing the high sensitivity and robustness of nuclear magnetic resonance (NMR) spectroscopy also uncovered that arginine methylation represents a highly abundant PTM and can be dynamically modulated by biological processes such as differentiation and aging.^4^ Collectively, the comprehensive methylome analyses revealed the existence of a vast number of arginine-methylated proteins, making it more difficult to attribute the impact of global changes in arginine methylation on physiological functions to the specific substrates.

Among the PRMT family, PRMT1 is the principal member responsible for as much as 85% of the cellular arginine methylation in mammals^5^ and regulates a wide variety of cellular processes, e.g., transcription, mRNA splicing, signal transduction, DNA repair, and immune system.^6^ Since the *Prmt1* complete knockout exhibits embryonic lethality in mice,^7^ the physiological significance of PRMT1 has been shown by the studies using tissue-specific conditional knockout (cKO) mice. We previously generated three cKO lines, each of which lacks PRMT1 in the central nervous system,^8^ cardiomyocyte,^9^ or endothelial cells,^10^ and demonstrated that PRMT1 is essential for oligodendrocyte maturation, cardiac development, and embryonic vascular formation, respectively. Furthermore, other groups have reported the crucial roles of PRMT1 in epigenetically mediated control of muscle regeneration,^11^ glucose homeostasis of the white adipose tissues,^12^ and the maintenance of intestinal epithelial architecture,^13^ by examining tissue-specific *Prmt1* cKO mice. However, given the ubiquitous expression of PRMT1, the cKO approach has limitations in elucidating “*bona fide*” functions of PRMT1 in the whole body. To move beyond loss-of-function approaches, there is a pressing need for models that can probe the consequences of enhanced PRMT1 activity throughout the entire organism. Previously, a conditional PRMT1 overexpression mouse model driven by tissue-specific promoters was developed to investigate the oncogenic roles of PRMT1 in epithelial tissues, particularly in mammary gland tumorigenesis.^14^ However, there is currently no mouse model expressing a hyperactive variant of PRMT1 under the control of the endogenous promoter, reflecting physiologically relevant expression patterns. Consequently, it remains unclear whether systemic enhancement of PRMT1 enzymatic activity can broadly influence physiological processes beyond specific tissues or cell types.

Notably, PRMT1 is extremely conserved across species, and indeed, only a single amino acid substitution at position 179 exists between mice (histidine, H) and humans (tyrosine, Y). Since this substitution is located outside the catalytic core domain of PRMT1, it was initially assumed to represent a neutral mutation that has no effect on protein function and organism fitness. However, in our evaluation of PRMT1 enzymes *in vitro*, we noticed that this seemingly inconsequential substitution between humans and mice could be involved in the difference in their catalytic activities. This unexpected finding led us to investigate here the functional impact of the H179Y substitution in PRMT1 at the molecular and organismal levels. In this study, by using both *in vitro* enzymatic assays and molecular dynamics (MD) simulations, we demonstrate that human PRMT1 exhibits higher methyltransferase activity than its mouse counterpart. To assess the *in vivo* consequences of the H179Y substitution, we generated humanized PRMT1 mice (huMice) possessing the H179Y substitution in the *prmt1* gene. Our knock-in mice represent a physiologically regulated gain-of-function model, allowing the investigation of systemically enhanced PRMT1 activity *in vivo*. Comprehensive RNA sequence (RNA-seq) transcriptomic profiling of these huMice clarified significant differences in the regulation of inflammatory responses in old age. Our study provides a direct link between PRMT1 activity and age-related physiology, such as inflammaging.

## Results

### A single amino acid distinguishes hPRMT1 from mPRMT1

**Figure 1:**
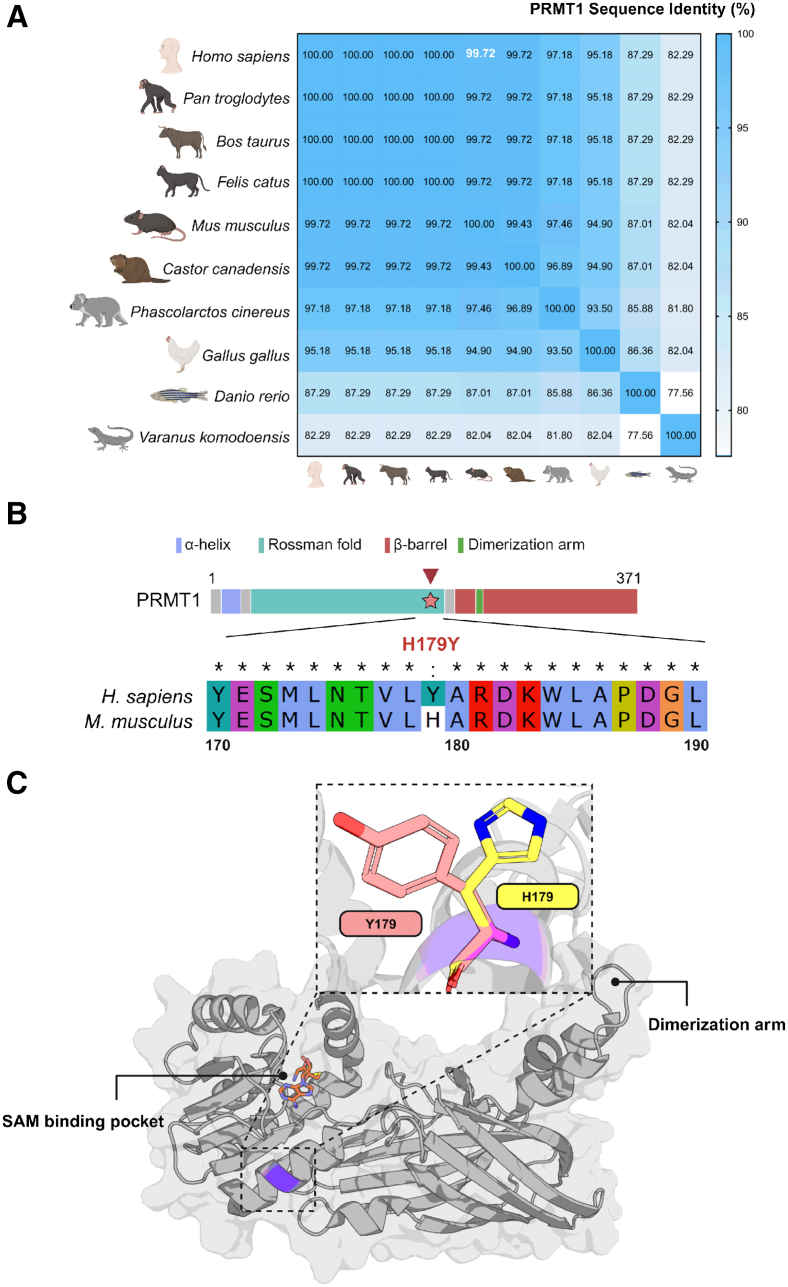
Sequence conservation and structural comparison between human and mouse PRMT1, highlighting the H179Y substitution (A) Heatmap representing the percentage of PRMT1 sequence identity across several species, with similarities ranging from low (white) to high (blue). Mouse and human PRMT1 show 99.72% sequence identity, highlighted in white. Created with BioRender.com. (B) Schematic representation and protein sequence alignment of human PRMT1 (hPRMT1) and mouse PRMT1 (mPRMT1) are illustrated by focusing on the permutation region (residues 170–190). The alignment reveals a single amino acid variation at position 179th, where human PRMT1 contains a tyrosine (Y) residue and mouse PRMT1 harbors a histidine (H). The top diagram illustrates the domain architecture of full-length PRMT1, with key domains and motifs labeled. The star symbol (★) and a red arrow indicate the position of the amino acid substitution (H179Y). The bottom panel shows an enlarged view of the aligned sequence region surrounding residue 179. Asterisks (∗) denote identical amino acids between human and mouse PRMT1; colon (:) indicates non-identical residues. (C) Secondary structure shown by the ribbon model of PRMT1 with the single amino acid variation between human and mouse orthologs is highlighted. Key functional regions, including the SAM binding pocket and dimerization arm, are highlighted to contextualize the mutation’s potential impact on PRMT1 function.

PRMT1 is a highly conserved enzyme across various species, with a remarkably 99.72% sequence similarity between human and mouse orthologs (Figure 1A). Even in the process of the continuous evolution of organisms, the amino acid sequence of PRMT1 remains largely unchanged, suggesting that the composition and sequence of the individual amino acid residues that make up the enzyme are indispensable for its authentic function. Intriguingly, despite this high degree of homology,^15^ a single amino acid variation distinguishes human (Y) and mouse (H) PRMT1 sequences at position 179 (Figure 1B). The position of the 179th residue is located neither within the SAM binding pocket, which is essential for the enzymatic activity^16^^,^^17^ nor in close proximity to the dimerization arm, a region crucial for PRMT1 dimerization and optimal enzymatic function^16^^,^^17^^,^^18^ (Figure 1C). Instead, it resides in a domain of unknown function, raising the intriguing possibility that this subtle evolutionary substitution could influence PRMT1’s enzymatic behavior or regulatory properties. Recognizing this unique difference sets the stage for further investigation into how a single amino acid change can alter PRMT1 function at the molecular and physiological levels.

### hPRMT1 contains higher methyltransferase activity compared to mouse PRMT1

**Figure 2:**
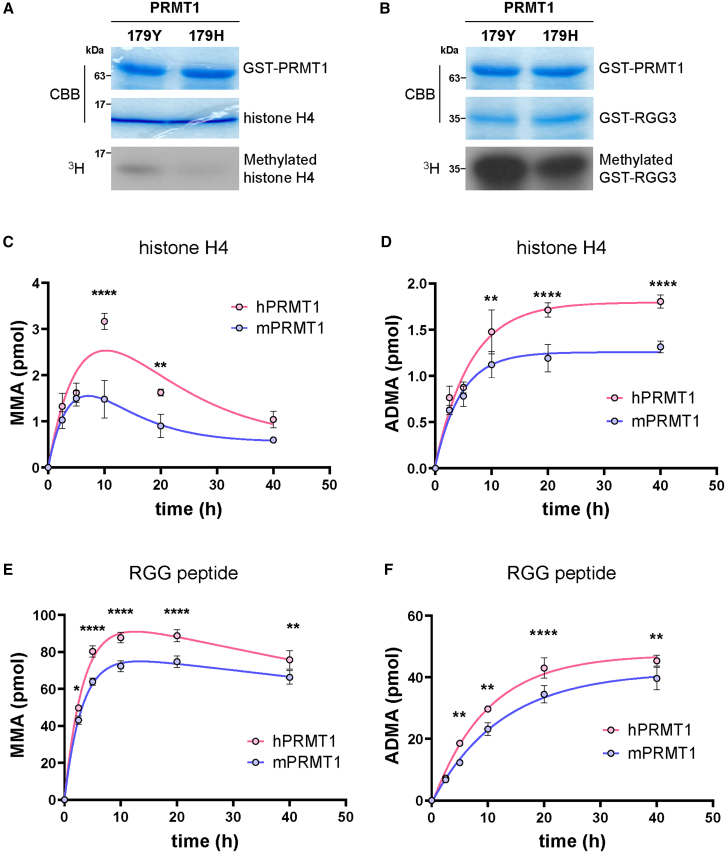
Comparison of *in vitro* methylation activity of hPRMT1 and mPRMT1 on histone H4 and EWS-RGG3 (A and B) *In vitro* methylation assay of histone H4 (A) and EWS RGG3 motif (B). Both substrates were incubated with purified GST-fused hPRMT1 or mouse PRMT1 in the presence of [^3^H] SAM at 30°C for 40 min. The methylation signals were detected by fluorography (down panel). Total amounts of recombinant histone H4 and GST-fused PRMT1 were determined by Coomassie brilliant blue (CBB) staining (upper panel). (C and E) Time course of MMA formation on the histone H4 (C) and EWS RGG peptide (E) catalyzed by human (red) or mouse (blue) PRMT1. The EWS RGG peptide was incubated with PRMT1 and SAM for the indicated times (2.5–40 h), and the reaction products were analyzed by LC-MS/MS to quantify MMA levels. Data are presented as mean ± SEM (*n* = 3 independent experiments). (D and F) Time course of ADMA formation on the histone H4 (D) and EWS RGG peptide (F) catalyzed by human (red) or mouse (blue) PRMT1. Data are presented as mean ± SEM (*n* = 3 independent experiments). Statistical significance was determined by two-way ANOVA with Sidak’s multiple comparisons test. ∗*p* < 0.05, ∗∗*p* < 0.01, ∗∗∗∗*p* < 0.0001.

To determine whether the difference of amino acid residue in position 179 of PRMT1 between human and mouse affects the enzymatic activity of PRMT1, we examined its activity on two canonical PRMT1 substrates, which are histone H4 and the RNA-binding protein EWS. Histone H4 methylation by PRMT1 modulates transcription, chromatin remodeling, and DNA repair,^19^^,^^20^^,^^21^ while EWS, harboring multiple RGG repeats in its C-terminal domain, serves as a representative RGG-type substrate.^22^^,^^23^^,^^24^^,^^25^ It should be noted that the sequences of histone H4 and the RGG region of EWS are identical between humans and mice, enabling a direct comparison of human (hPRMT1) and mouse (mPRMT1) enzymes under identical conditions. First, we performed *in vitro* methylation assays using radiolabeled SAM. Recombinant histone H4 and GST-fused EWS RGG3 motif (residues 543–656) were incubated with hPRMT1 or mPRMT1 in the presence of [^3^H] SAM at 30°C for 40 min. The methylation signals were then detected by fluorography, revealing that hPRMT1 exhibited a higher capacity for methylating both histone H4 and EWS RGG3 compared to mPRMT1 (Figures 2A, 2B, S1A–S1C).

To quantify these differences more precisely, we monitored the time-dependent formation of MMA and ADMA via LC-MS/MS. Recombinant histone H4 and EWS RGG3 peptide (see sequence in Figure S1D) were incubated with hPRMT1 or mPRMT1 at 30°C for various time points ranging from 2.5 to 40 h. Under identical reaction conditions, both substrates exhibited a similar pattern: MMA levels rose rapidly and plateaued within approximately 5 h, followed by slower ADMA accumulation, which reached saturation around 25 h (Figures 2C–2F). Notably, throughout this time course, hPRMT1 consistently produced higher levels of both MMA and ADMA than mPRMT1. These findings confirm that the single amino acid substitution endows hPRMT1 with enhanced methyltransferase activity toward key PRMT1 substrates. To quantitatively define the observed enzymatic differences between hPRMT1 and mPRMT1, we performed detailed steady-state kinetic analyses using the EWS RGG3 peptide substrate. Initial reaction rates for MMA and ADMA formation were measured using LC-MS/MS, varying peptide concentrations from 0 to 100 μM under saturating SAM conditions (50 μM). Michaelis–Menten kinetic parameters (V_max_, K_m_, k_cat_, and catalytic efficiency k_cat_/K_m_) were derived from these experiments (Figure S1E, Table S1). For MMA formation, hPRMT1 exhibited a slightly higher catalytic turnover (k_cat_) and catalytic efficiency (k_cat_/K_m_) compared to mPRMT1 (k_cat_: 15.82 × 10^−2^·min^−1^ vs. 14.78 × 10^−2^·min^−1^; k_cat_/K_m_: 1.92 × 10^−2^ ·min^−1^·μM vs. 1.86 × 10^−2^ ·min^−1^·μM). A similar trend was observed for ADMA formation, with hPRMT1 displaying enhanced catalytic parameters relative to mPRMT1 (k_cat_: 0.77 × 10^−2^·min^−1^ vs. 0.61 × 10^−2^·min^−1^; k_cat_/K_m_: 0.48 × 10^−2^ ·min^−1^·μM vs. 0.32 × 10^−2^ min^−1^·μM), although both enzymes exhibited substantially lower V_max_ values for ADMA formation, consistent with PRMT1’s known distributive dimethylation mechanism. These findings reinforce the conclusion that the single amino acid substitution at position 179 (His in mouse, Tyr in human) fine-tunes the catalytic properties of PRMT1, potentially enhancing substrate recognition or catalytic efficiency toward specific substrates.

To further investigate whether the enhanced methylation activity of hPRMT1 applies broadly or is substrate-specific, we evaluated FOXO1. PRMT1 has been reported previously to methylate FOXO1 preferentially at Arg248 and Arg250.^26^ We thus conducted methylation assays utilizing recombinant mouse FOXO1 fragment (amino acids 37–338) as a substrate, focusing specifically on the highly conserved arginine-containing sequence (246-SPRRRAASMD-255), identical between mouse and human FOXO1. Recombinant mFOXO1 fragment (residues 37–338) was incubated with hPRMT1 or mPRMT1 at 30°C for 6 h, and methylation was quantified by LC-MS/MS. Interestingly, no significant difference in methylation was observed between hPRMT1 and mPRMT1 for FOXO1 (Figure S1F), suggesting the enhanced catalytic activity conferred by the H179Y substitution is substrate-dependent and does not uniformly extend to all PRMT1 targets.

### hPRMT1-RGG-SAM complex shows enhanced stability compared to mPRMT1

**Figure 3:**
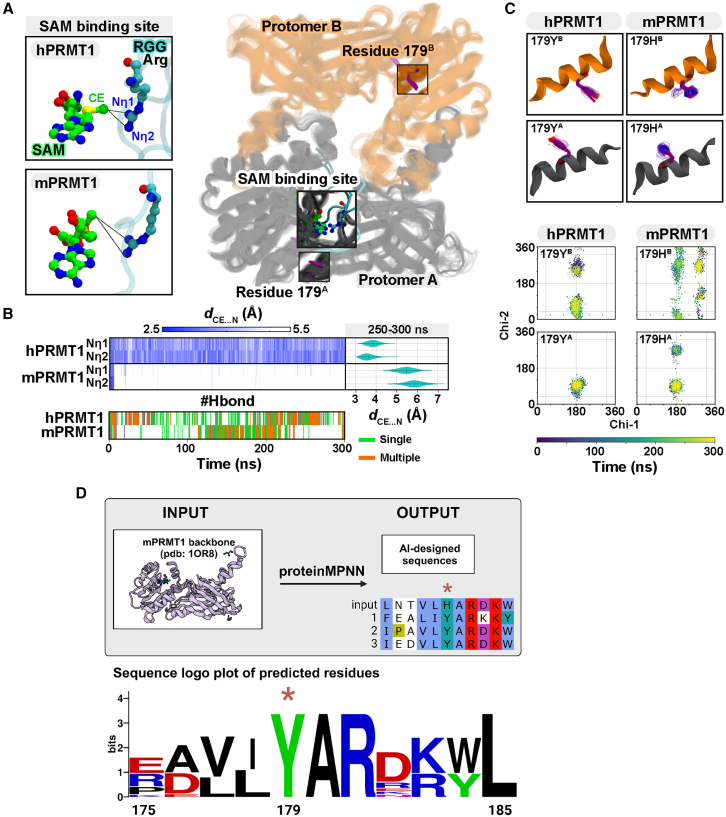
Molecular dynamics simulation of SAM-binding and substrate interaction in hPRMT1 and mPRMT1 (A) Structural representation of the dimeric PRMT1 focusing on the SAM-binding site and the interaction between the CE atom of SAM and the guanidino group of the substrate arginine during MD simulations. Protomers A and B are represented in gray and orange. The upper panel shows the hPRMT1 model and the bottom panel shows the mPRMT1 model. Atoms are represented as ball-and-stick. (B) The heatmap plots a comparison of the *d*_CE … N_ and #Hbond between hPRMT1 and mPRMT1 throughout the 300 ns-MD simulation. The right violin plot shows the frequency of the *d*_CE … N_ focusing on the last 50 ns. Hbond analysis in the below panel measured between SAM and nearby residues. Green indicates a single hydrogen bond, and orange represents multiple hydrogen bonds. (C) The side chain torsional dynamics of the 179th residue of PRMT1 is illustrated by 3D structure and Chi1-Chi2 plot. (D) The upper panel illustrates the prediction workflow, where the backbone structure of mPRMT1 (PDB: 1OR8) was used as input for 15 independent runs of ProteinMPNN. The analysis specifically focused on the predicted amino acid at position 179. The lower panel shows a sequence logo plot highlighting the frequency of predicted amino acids from positions 175 to 185. Remarkably, position 179 was consistently predicted as tyrosine across all runs, aligning with the sequence found in human PRMT1. The asterisks (∗) indicates the position of the amino acid substitution (179).

Given the observed enhancement in hPRMT1 enzymatic activity, we next explored how the H179Y substitution influences substrate recognition at an atomic level. Using molecular dynamics (MD) simulations, we analyzed PRMT1–SAM–RGG complexes (employing the same EWS-derived RGG sequence as in Figure 2) to assess how this single amino acid change impacts the conformational landscape. Arginine methylation involves the transfer of a methyl group from SAM to a nitrogen atom in the guanidino group of an arginine residue within a substrate. Since epsilon carbon of methyl group (CE) in SAM is electrophilic and can accept nucleophilic attacks from groups like the nitrogen in the guanidino group of arginine, to evaluate the stability of the binding interaction, we measured the distance between the CE atom of SAM and the nitrogen of the guanidino group (Nη) in the arginine of the RGG substrate (*d*_CE … N_). Over a 300 ns-MD trajectory (with three independent replicates), hPRMT1 consistently maintained a stable geometry conducive to methyl transfer, whereas mPRMT1 rapidly lost this alignment (Figure 3A). At the last 50 ns of MD trajectories (Figure 3B), the distances between the methyl group of SAM and the Nη1 and Nη2 atoms in hPRMT1 (3.84 ± 0.28 Å and 3.60 ± 0.28 Å) confirmed a closer and more stable interaction compared to mPRMT1 (5.46 ± 0.37 Å and 5.87 ± 0.43 Å). Hydrogen bonding (Hbond) analysis between SAM and nearby residues in the hPRMT1 systems showed higher stability compared to the mPRMT1 systems over the 300 ns-MD trajectories (Figure 3B). In hPRMT1, SAM was consistently stabilized by Hbond with E147 (68.00%) and H63 (27.00%), whereas in mPRMT1, SAM formed a hydrogen bond with E147 (33.00%) and a weaker interaction with H63 (11.00%).

This significant difference might be caused by the variation in the side chain dynamics between Y (hPRMT1) and H (mPRMT1) at position 179. Thus, we examined the side chain dihedral angles of Y179 and H179, focusing on protomer A with the RGG substrate bound to the SAM binding site (Figure 3C). The tyrosine variant adopted a more restricted conformational space, stabilizing the substrate-binding region, whereas histidine displayed greater torsional flexibility and disrupted optimal substrate positioning (Figure 3C). The fluctuation of this histidine may affect the amino acid communication, leading to the loss of interaction with the guanidino group of arginine in the RGG substrate. Outside the substrate-bound protomer, both Y179 and H179 showed more variable conformations, indicating that the substrate itself helps define the residue’s structural constraints. To further investigate the effect of this substitution on structural integrity, we performed residue interaction network analysis using the Residue Interaction Network Generator (RING). The analysis revealed that Y179 in hPRMT1 forms a network of stabilizing interactions with key residues near the SAM-binding site, including E147, T176, D182, and K183 (Figure S2A). These interactions are essential for maintaining the geometry and electrostatics of the catalytic pocket. In contrast, H179 in mPRMT1 exhibited diminished and fewer interactions, especially lacking the hydroxyl group and π-stacking capacity of tyrosine, interacting only weakly with E147 and T176. This loss of connectivity likely contributes to reduced catalytic preorganization and explains the diminished enzymatic activity in mPRMT1.

To probe the structural implications of the H179Y substitution, we then employed ProteinMPNN,^27^ a computational protein design algorithm that optimizes amino acid sequences for improved folding and stability.^27^^,^^28^ When supplied with the backbone structure of mPRMT1 (H at position 179), ProteinMPNN consistently selected tyrosine at this position across all fifteen independent iterations (Figures 3D and S2B). This repeated prediction suggests that tyrosine, rather than histidine, is more compatible with the structural framework of PRMT1, potentially enhancing local packing, hydrogen bonding, and overall conformational stability. While these predictions require experimental validation, they provide a strong computational indication that the H179Y substitution confers structural advantages aligned with the observed increase in the catalytic activity of hPRMT1.

### huMice MEFs confer increased resistance to inflammatory stress

**Figure 4:**
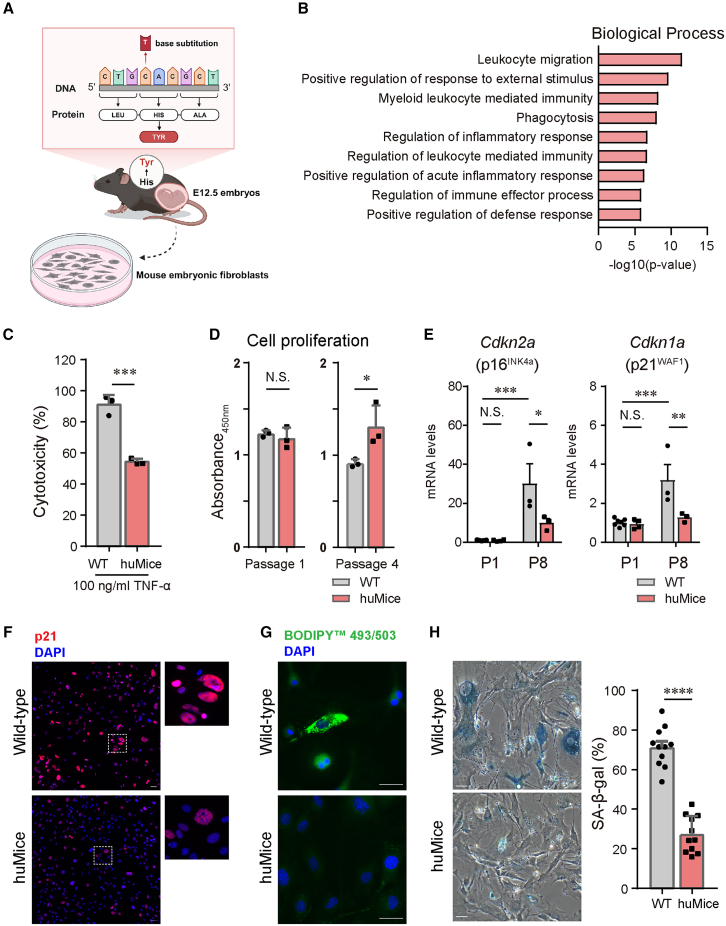
Generation and phenotypic characterization of humanized PRMT1 mice (huMice) and cellular analyses of MEFs (A) Schematic representation of the targeted replacement of histidine (H) at position 179 of mouse PRMT1 with tyrosine (Y), resulting in the generation of huMice. This panel also depicts the approach employed to derive mouse embryonic fibroblasts (MEFs) from E12.5 huMice embryos, enabling subsequent *in vitro* analyses. Created with BioRender.com. (B) Gene ontology (GO) enrichment analysis of differentially expressed genes (DEGs) identified between male MEFs from WT and huMice. (C) TNF-α–induced cytotoxicity in male MEFs from WT and huMice. MEFs at passage 8 were exposed to 100 ng/mL TNF-α for 24 h, and cytotoxicity (%) was determined by measuring LDH release according to the manufacturer’s instructions. Data are presented as mean ± SEM (*n* = 3 independent MEF preparations). Statistical analysis was performed using unpaired *t*-test. ∗∗∗*p* < 0.001. (D) Proliferation analysis in male MEFs from WT and huMice at passage 1 (left) and passage 4 (right) using the CCK-8 assay. MEFs were seeded in 96-well plates and incubated for 24 h, after which absorbance at 450 nm was measured to assess cell number. Data are presented as mean ± SEM (*n* = 3 independent MEF preparations). Statistical analysis was performed using unpaired two-tailed *t*-test. N.S., no significant differences. ∗*p* < 0.05. (E) Expression levels of *Cdkn2a*/p16^Ink4a^ and *Cdkn1a*/p21^WAF1^ in male MEFs at passage 1 and passage 8. At passage 1, *n* = 7 (WT) and *n* = 4 (huMice); at passage 8, *n* = 3 per group. Data are presented as mean ± SEM. Statistical analysis was performed using two-way ANOVA followed by Tukey’s multiple comparisons test. *N.S.*, no significant differences. ∗*p* < 0.05, ∗∗*p* < 0.01, ∗∗∗*p* < 0.001. (F) Representative images of male MEFs at passage 6 stained with antibodies against p21 and DAPI. Scale bars, 50 μm. (G) Representative images of lipid droplet staining with BODIPY 493/503 in male MEFs. Scale bars, 50 μm. (H) Representative images of senescence-associated β-galactosidase (SA-β-gal) staining in late-passage male MEFs from WT and huMice. Quantification shows the percentage of SA-β-gal–positive cells, based on *n* = 11 microscopic fields per group: WT (pooled from three independent MEFs, passage 11) and huMice (pooled from six independent MEFs, passage 12). Data are presented as mean ± SEM. Statistical analysis was performed using unpaired two-tailed *t*-test. ∗∗∗∗*p* < 0.0001. Scale bars, 50 μm.

To explore the physiological consequences of this substitution, we generated a genetically modified mouse model, termed huMice, in which the H179 of mPRMT1 was replaced with tyrosine (Figure 4A). We confirmed that the mRNA expression levels of the two major Prmt1 isoforms, variant 1 (V1) and variant 2 (V2), were comparable between wild-type (WT) mice and huMice in heart, kidney, cerebrum, and cerebellum tissues at 2 months of age, indicating that the H179Y substitution does not affect PRMT1 isoform expression *in vivo* (Figures S3A–S3D). Moreover, huMice exhibited no phenotypic abnormalities, with body and organ weights comparable to WT mice from birth (Figures S3E–S3I). To investigate the cellular effects of this substitution, we isolated mouse embryonic fibroblasts (MEFs) from E12.5 embryos (Figure 4A). Since PRMT1 plays an important role in transcription by methylating histone H4 and various transcription factors,^2^ we performed RNA-seq analysis on MEFs to determine the effects of the H179Y substitution on the gene expression. Gene Ontology (GO) analysis revealed significant enrichment of differentially expressed genes (DEGs) related to inflammatory responses, notably “Positive regulation of acute inflammatory response” and “Regulation of inflammatory response” (Figure 4B). These findings suggest an altered inflammatory response capacity in huMice MEFs.

To validate these transcriptomic findings, we assessed the inflammatory response by treating MEFs with tumor necrosis factor α (TNF-α), a potent inducer of cell death, and measured the lactate dehydrogenase (LDH) release as an indicator of cytotoxicity. huMice MEFs exhibited lower LDH release compared to WT MEFs following TNF-α treatment (Figure 4C), confirming that hPRMT1 enhances cellular resistance to inflammatory stress. Chronic inflammatory signaling is closely associated with cellular senescence, an important stress response program implicated in embryonic development, aging, and immunity.^29^ We thus investigated whether huMice MEFs display altered senescence profiles under prolonged culture conditions. Consistent with enhanced stress resistance, proliferation rates were comparable at early passages, whereas huMice MEFs significantly outperformed WT controls at later passages in a 24-h proliferation assay (Figure 4D). Supplementary data confirmed that huMice MEFs consistently maintained enhanced proliferative capacity over extended culture periods (Figure S4A).

We further investigated whether huMice MEFs exhibit additional hallmarks of cellular senescence in prolonged cultures, adhering to recently established comprehensive guidelines.^30^ First, we analyzed the transcript levels of the key cell-cycle inhibitors *Cdkn2a* (encoding p16^INK4a^) and *Cdkn1a* (encoding p21^WAF1^), canonical markers indicative of senescence-associated cell-cycle arrest. Quantitative RT-PCR revealed that at an early passage (passage 1), expression levels of *Cdkn2a* and *Cdkn1a* were comparable between WT and huMice MEFs (Figure 4E). Primer sequences used for qRT-PCR are listed in Table 1. However, at a later passage (passage 8), WT MEFs showed significantly elevated expression of both genes, whereas huMice MEFs maintained lower expression levels (Figure 4E). These findings suggest delayed activation of the senescence-associated transcriptional program in huMice MEFs. To validate transcriptional observations at the protein level, immunofluorescence staining of p21 was performed in late-passage cells. In line with our qPCR data, WT MEFs exhibited robust p21 protein level, indicative of pronounced cell-cycle arrest and senescence onset, whereas huMice MEFs showed notably weaker staining intensity (Figure 4F), further substantiating the transcriptional data.

**Table 1:** Primer sequences used for quantitative RT-PCR, related to STAR Methods

Gene	Forward primer (5'→3′)	Reverse primer (5'→3′)
Tnf	AATGGCCTCCCTCTCATCAG	GCTACGACGTGGGCTACAGG
Il6	CTCTGCAAGAGACTTCCATCCAGT	CGTGGTTGTCACCAGCATCA
Prmt1 variant1	TGCATCATGGAGGTTTCCTGT	CATCCTTCAGCATCTCCTCGT
Prmt1 variant2	GAACTGCATCATGGAGAATTTTG	TGGCCACAGGAAACTTCTTC
Cdkn2a (p16)	GCTCAACTACGGTGCAGATTC	GCACGATGTCTTGATGTCCC
Cdkn1a (p21)	CGAGAACGGTGGAACTTTGAC	CCAGGGCTCAGGTAGACCTT

Cellular senescence is also accompanied by marked metabolic alterations, notably an increase in intracellular lipid accumulation.^30^ To assess this hallmark, we employed BODIPY 493/503 staining to visualize lipid droplets. WT MEFs at late passage displayed significantly enhanced lipid accumulation, whereas huMice MEFs demonstrated a reduced lipid droplet load (Figure 4G). This differential lipid accumulation provides additional evidence supporting delayed metabolic senescence in huMice MEFs. Supporting these findings, quantitative analysis of senescence-associated β-galactosidase (SA-β-gal) activity further confirmed a notably reduced frequency of SA-β-gal-positive cells in huMice MEFs at late passages (Figure 4H). Collectively, these findings from transcriptomic profiling, protein-level validation, and metabolic assessment demonstrate that huMice MEFs exhibit enhanced resilience to inflammatory stress and significantly attenuated senescence phenotypes, implicating the H179Y substitution in PRMT1 as a modulator of cellular aging responses.

### huMice show age-dependent transcriptional divergence in inflammatory responses

**Figure 5:**
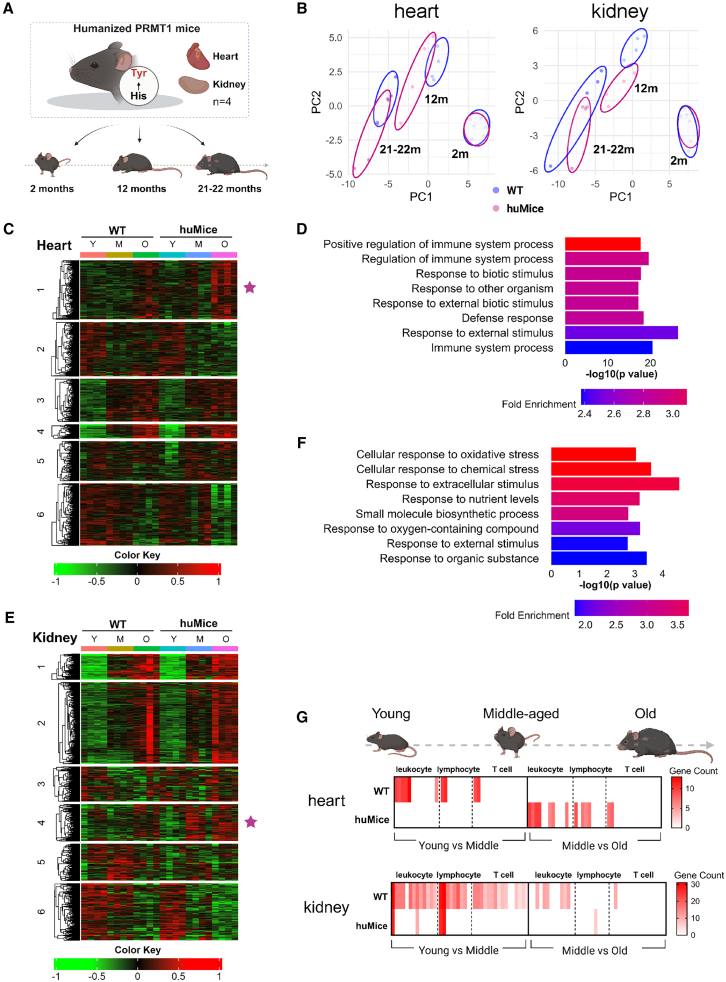
RNA-seq and GO analysis of inflammation-related transcriptional changes in huMice across different ages (A) Schematic representation of the RNA-seq strategy. Heart and kidney tissues from male WT and huMice at 2, 12, and 21–22 months of age were subjected to RNA-seq analysis. Created with BioRender.com. (B) Principal component analysis (PCA) of the RNA-seq data from heart (left) and kidney (right) tissues of WT mice (blue circles) and huMice (red circles) at different ages. Each point represents a single sample. (C and E) RNA-seq data from heart and kidney tissues of male WT and huMice at 2, 12, and 21–22 months were VST-normalized and subjected to k-means clustering analysis, resulting in 6 distinct clusters. The heatmap represents the centered and scaled expression levels for the top 2,000 genes ranked by standard deviation across all samples. Each gene’s expression level was centered by subtracting its average expression across all samples. Clusters associated with stress response are indicated with star symbols. For clarity, age groups are labeled as Y (young, 2 months), M (middle-aged, 12 months), and O (old, 21–22 months). (D and F) Gene Ontology (GO) analysis was performed for the clusters with star symbol identified in the k-means clustering of RNA-seq data shown in (C, E). These clusters—Cluster 1 in the heart and Cluster 4 in the kidney—were enriched for GO terms related to stress and stimulus responses, such as response to stress and stimulus. (G) Heatmaps showing the enrichment of GO terms related to T-cell-, lymphocyte-, and leukocyte-mediated immunity across different age groups in hearts (top) and kidneys (bottom). The color gradient reflects the number of genes contributing to each GO term, with darker colors indicating a greater number of genes. Comparisons are shown for two key age intervals: young (2 months) to middle-aged (12 months) (left), and middle-aged (12 months) to old (21–22 months) (right). These results highlight age-dependent transcriptional changes in immune-related processes. Created with BioRender.com.

Cellular senescence is thought to be one of the reasons for tissue age and the accumulation of senescent cells promotes enhanced biological aging and age-related diseases.^31^ To determine whether the cellular phenotype observed in huMice MEFs extends to the organismal level, we analyzed RNA-seq data from heart and kidney tissues of WT mice and huMice at 2, 12, and 21–22 months of age (Figure 5A). The choice of these two organs was based on our observation that huMice exhibited an increasing trend in ADMA levels, suggesting potential transcriptional differences mediated by hPRMT1 in these tissues (Figures S4B and S4C). At 2 months, principal component analysis (PCA) revealed minimal transcriptional differences between WT mice and huMice, whereas their gene expression profiles diverged markedly as the mice aged (Figure 5B), indicating a progressive, age-dependent impact of the H179Y substitution on global gene regulation. To investigate how tyrosine 179 residue in the PRMT1 affects age-related transcriptional changes, we defined an “age-associated gene set” from publicly available transcriptomic data spanning multiple organs and ages.^32^ Gene Set Enrichment Analysis (GSEA) revealed enrichment of this gene set in heart and kidney tissues from 21 to 22-month-old WT mice, whereas huMice tissues showed reduced enrichment (Figure S4D). These findings suggest that hPRMT1 may confer resistance to age-related transcriptional changes.

Building on these observations, we conducted a more detailed analysis to examine age-related transcriptional changes within each genotype. We next applied k-means clustering to all RNA-seq data from WT mice and huMice hearts and kidneys (Figures 5C and 5E).^33^ Early in life (2 months), both genotypes showed similar expression patterns for stress- and immune-related gene clusters. However, by 12 and 21–22 months, these clusters diverged in huMice, revealing distinct regulation of genes involved in responses to external stimuli, defense mechanisms, and oxidative stress compared to WT mice (Figures 5C–5F). Thus, while young mice share comparable transcriptional profiles, aging leads huMice to adopt an altered, potentially more controlled, inflammatory, and stress response program. Consistent with this trend, differential expression analyses linked to aging uncovered limited overlap in age-responsive genes between WT mice and huMice (Figure S5A). GO enrichment analysis of DEGs between the start (2 months) and end (21–22 months) points of hearts and kidneys highlighted immune cell activation and inflammation, reflecting the broad impact of aging on inflammatory responses in both genotypes (Figures S5B and S5C). Notably, these immune-related changes occurred prominently earlier in WT mice hearts (2–12 months), while in huMice, they were delayed and reduced (Figure 5G). Similar patterns emerged in the kidneys, where WT mice sustained inflammatory gene expression changes from 2 to 21–22 months, whereas huMice showed fewer such alterations (Figures 5G and S5C). By 12 months—a critical time point for this transcriptional divergence—huMice hearts already differed from WT mice in their response to cytokines and stress stimuli (Figure S5D). Collectively, these data establish that hPRMT1 modifies age-dependent transcriptional trajectories and attenuates the inflammatory signatures that typically emerge with aging. By 12 months, huMice begin to exhibit a distinct, less inflammatory transcriptional profile, underscoring the role of hPRMT1 in modulating aging-related inflammation.

### huMice exhibit resistance to LPS-induced inflammatory stress

**Figure 6:**
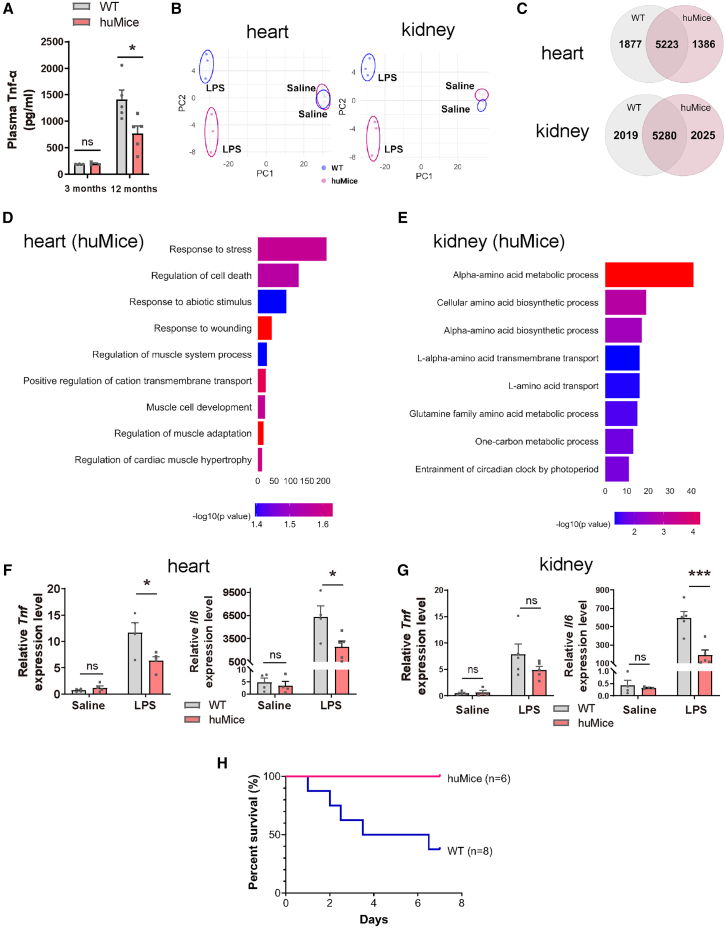
Differential inflammatory responses in WT mice and huMice following LPS challenge (A) Plasma tumor necrosis factor (TNF) levels in male WT and huMice 4 h after LPS injection in both 3-month-old (left, *n* = 3) and 12-month-old (right, *n* = 5) mice, measured by ELISA. While no differences were observed at 3 months, huMice showed lower TNF levels compared to WT mice at 12 months. Data are mean ± SEM. Statistical analysis was performed using two-way ANOVA followed by Tukey’s multiple comparisons test. ns, no significant differences. ∗*p* < 0.05. (B) Principal component analysis (PCA) of RNA-seq data from heart (left) and kidney (right) tissues of male WT and huMice (12 months) before and after LPS treatment. Saline-treated controls are also shown. (C) Venn diagram illustrating the distribution of DEGs in male WT and huMice following LPS stimulation. The overlapping region represents the shared DEGs between WT and huMice, while the non-overlapping sections represent the unique DEGs for WT and huMice in both heart and kidney tissues. (D, E) GO analysis of huMice-specific unique DEGs identified in the Venn diagram (Figure 6C). (D) In the heart, huMice-specific DEGs were enriched for terms related to response to stress, regulation of cell death, and regulation of cardiac muscle hypertrophy. (E) In the kidney, huMice-specific DEGs showed enrichment in GO terms associated with amino acid metabolic process and L-amino acid transport, providing further insight into the transcriptional divergence induced by the H179Y substitution. (F) mRNA expression levels of *Tnf* and *Il6* in the hearts of male WT and huMice 4 h after LPS injection for 12-month-old age group, as assessed by quantitative real-time PCR (qRT-PCR). Saline-treated controls are included (*n* = 4–5 per group). Data are mean ± SEM. Statistical analysis was performed using two-way ANOVA followed by Tukey’s multiple comparisons test. ns, no significant differences. ∗*p* < 0.05. (G) mRNA expression levels of *Tnf* and *Il6* in the kidneys of male WT and huMice 4 h after LPS injection for 12-month-old age group, as assessed by qRT-PCR. Saline-treated controls are included (*n* = 4–5 per group). Data are mean ± SEM. Statistical analysis was performed using two-way ANOVA followed by Tukey’s multiple comparisons test. *ns*, no significant differences. ∗∗∗*p* < 0.001. (H) Survival curves of male WT and huMice following lipopolysaccharide (LPS) injection (2.0 mg/kg, i.p.). Survival was monitored for 7 days post-injection.

Based on the results of transcriptome analysis, we subjected 12-month-old mice to LPS challenge, a potent stimulus for systemic inflammation,^34^ to investigate whether the transcriptional differences observed in huMice translate into physiological resilience. We compared the inflammatory response of both 3-month-old and 12-month-old huMice and WT mice to assess whether the observed inflammatory resistance in huMice is age-dependent. At 3 months of age, there was no difference in plasma TNF-α levels between huMice and WT mice 4 h post-LPS treatment, indicating a similar inflammatory response at this early stage (Figure 6A). However, by 12 months, WT mice exhibited an age-related increase in plasma TNF-α levels, reflecting the age-related increase in inflammatory responses, commonly referred to as inflammaging (Figure 6A). In contrast, 12-month-old huMice displayed markedly lower plasma TNF-α levels compared to WT mice, indicating a suppression of the age-related inflammatory response (Figure 6A). To understand the transcriptional basis underlying the enhanced resistance to LPS in huMice, we performed RNA-seq analysis on hearts and kidneys following LPS treatment. PCA showed that while WT mice and huMice clustered similarly under saline conditions, their responses to LPS diverged markedly, with hPRMT1 altering the transcriptional landscape in a distinct direction (Figure 6B). GO analysis between LPS-treated WT mice and huMice, showing enrichment of gene expressions in response to LPS and regulation of inflammatory response in both heart and kidney (Figure S6A).

To gain deeper insight, we compared DEGs between WT mice and huMice under saline-to-LPS stimulation, identifying 5,223 shared DEGs in the heart and 5,280 in the kidney. Beyond these shared changes, each genotype also exhibited unique DEGs: WT mice had 1,877 and 2,019 unique DEGs in the heart and kidney, respectively, while huMice showed 1,386 and 2,025 unique DEGs (Figure 6C). Building on these differences, we further examined the unique DEGs that emerged specifically in response to LPS in each genotype. In WT mice, these WT-specific DEGs were enriched for pathways related to DNA replication and repair (Figures S6B and S6C). While these processes are essential for maintaining genomic integrity and repairing tissue damage under stress conditions,^35^ their excessive activation under systemic inflammatory stress may deplete cellular resources or exacerbate replication stress, ultimately compromising tissue function.^36^ In contrast, huMice-specific DEGs were enriched for GO terms related to “response to stress,” “regulation of cell death,” and “amino acid metabolic processes” (Figures 6D–6E). These findings suggest that huMice engage transcriptional programs focused on mitigating inflammatory damage and adapting to metabolic demands rather than overcommitting to DNA replication and repair.

To assess the expression of pro-inflammatory cytokines in various organs, we also performed qRT-PCR analysis of TNF and interleukin-6 (IL-6), two key pro-inflammatory cytokines,^37^^,^^38^ in the hearts, kidneys, bone marrow, and spleens at 4 h post-LPS injection. Primer sequences used for qRT-PCR are listed in Table 1. As shown in Figures 6F, 6G, S6D and S6E, LPS administration led to marked upregulation of *Tnf* and *Il6* expression across all tissues examined in WT mice, but this upregulation was significantly diminished in huMice, confirming the protective effect of hPRMT1 on LPS-induced inflammatory response. In addition, MEFs derived from huMice also demonstrated minimal *Tnf* and *Il6* induction in response to LPS, unlike WT MEFs that mounted a strong cytokine response (Figure S6F). These findings indicate that huMice engage distinct transcriptional programs in response to inflammatory stimuli, which may contribute to their enhanced resistance to LPS-induced stress and inflammation compared to WT mice. LPS-induced systemic inflammation causes multi-tissue damage and leads to the death.^39^^,^^40^ Importantly, while 37.5% of WT mice survived within a week of LPS administration, all huMice survived (100% survival rate, Figure 6H). Taken together, these findings suggest that the H179Y substitution in PRMT1 confers resistance to inflammatory stress at both the cellular and organismal levels, resulting in protection from organ failure and high mortality.

### hPRMT1 establishes distinct gene regulatory networks enriched for inflammatory response and stress pathways

**Figure 7:**
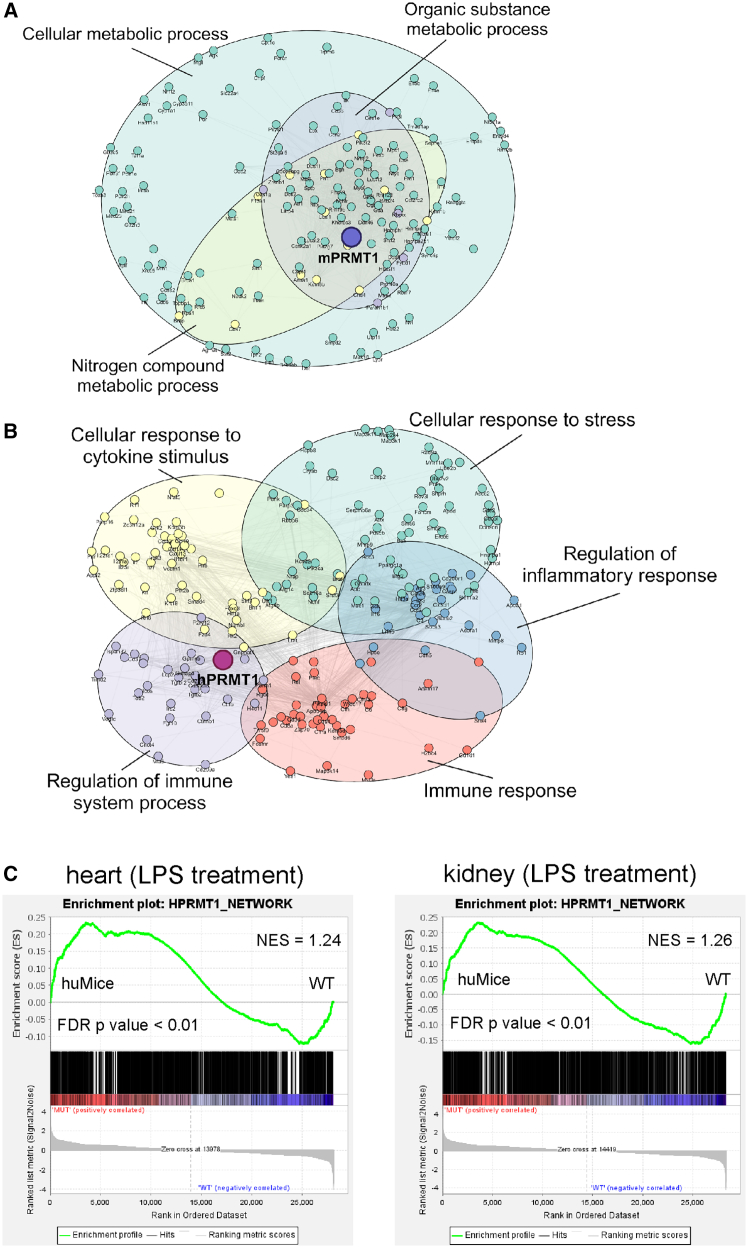
Gene network analysis of PRMT1 in WT mice and huMice, highlighting inflammatory stress response pathways associated with hPRMT1 (A) Gene network associated with mPRMT1 based on weighted gene co-expression network analysis (WGCNA) and STRING analysis. All RNA-seq data from heart and kidney tissues across ages (2, 12, and 21–22 months) in WT mice were used to construct mPRMT1-related modules via WGCNA. Genes within the mPRMT1-associated module were further analyzed using STRING to form a biologically meaningful network. The resulting network was associated with metabolic processes, including organic substance metabolic process and nitrogen compound metabolic process, as indicated by the absence of enriched inflammation or stress-related GO terms. (B) Gene network associated with hPRMT1 based on WGCNA and STRING analysis. Similarly, RNA-seq data from huMice heart and kidney tissues across 2, 12, and 21–22 months were used to identify hPRMT1-related modules. STRING analysis revealed that the hPRMT1-associated network was highly enriched for immune response-related functions, including regulation of inflammatory response, cellular response to stress, and response to cytokine stimulus, as indicated by distinct background colors for each enriched GO term. (C) Gene Set Enrichment Analysis (GSEA) of the hPRMT1-associated gene set identified from WGCNA modules (Figure 7B) under LPS stimulation. RNA-seq data from heart (left) and kidney (right) tissues were analyzed, revealing enrichment of the hPRMT1-associated gene set in both organs of huMice (FDR <0.01). Normalized enrichment scores (NES) were 1.24 for the heart and 1.24 for the kidney, underscoring the hPRMT1 network’s active involvement in transcriptional responses to inflammatory stress.

Building on the observed phenotypic differences, we leveraged WGCNA^41^ to dissect the systemic gene regulatory networks influenced by hPRMT1. While mPRMT1-related networks emphasized metabolic processes, hPRMT1 established highly interconnected modules enriched for “regulation of inflammatory response,” “cellular response to stress,” and “response to cytokine stimulus” across both heart and kidney at all ages examined (Figure 7A and 7B). This consistent enrichment underscores hPRMT1’s broad and systemic role in regulating transcriptional programs that govern inflammatory and stress responses, beyond its traditional involvement in metabolic regulation. Such extensive network integration by hPRMT1 likely contributes to the phenotypic differences observed in huMice, particularly their enhanced resistance to inflammatory stress.

To further validate the biological relevance of these hPRMT1-associated networks under inflammatory challenge, we generated an hPRMT1-specific inflammatory response gene set and applied GSEA to RNA-seq data from LPS-treated tissues. The hPRMT1 network was significantly upregulated in huMice hearts and kidneys following LPS stimulation (FDR *p* < 0.01, Figure 7C), aligning closely with the WGCNA findings. This alignment confirms that hPRMT1 not only correlates with but actively contributes to adaptive transcriptional responses under inflammatory stress. These findings provide strong evidence that the H179Y substitution drives a functional shift in PRMT1’s regulatory scope, linking its transcriptional influence to the phenotypic resilience observed in huMice.

## Discussion

To date, previous studies have predominantly focused on the loss-of-function effects of PRMT1, which have underscored its indispensable role in various tissues.^8^^,^^9^^,^^10^^,^^11^^,^^12^^,^^13^ These results collectively indicate that PRMT1 is crucial for maintaining tissue homeostasis and function, with its absence leading to physiological defects including pathological features. In this study, we moved beyond loss-of-function approaches to reveal how a single amino acid substitution (H179Y) in PRMT1 can reshape its enzymatic activity and broaden its systemic regulatory scope. In contrast, our present study established a modified model of PRMT1 by replacing His-179 with tyrosine, which substitution has been incidentally found to have enhanced catalytic activity of PRMT1. Unlike previous loss-of-function models, huMice display aging-associated phenotypes and the distinct gene regulatory networks involved in inflammatory and stress responses. This model will be an important tool for investigating the relationship between PRMT1 activity and function at the whole-body level, beyond the traditional analysis of tissue-specific *prmt1* knockout.

Based on the MD simulations, the hPRMT1 protein reveals a closer and more stable interaction between SAM, a methyl group donor, and the guanidino group of substrate arginine, supported by extended hydrogen bonding, potentially enhancing enzymatic activity (Figure 3). The H179Y substitution stabilizes the SAM residing in its position relative to arginine, as tyrosine exhibits reduced side-chain fluctuations (chi1 and chi2) compared to histidine, promoting consistent substrate alignment for efficient methyl transfer (Figure 3). This analysis highlights how single amino acid changes can reshape the enzyme microenvironment and dynamics, driving functional divergence in homologous enzymes. Previous kinetic studies have primarily focused on human PRMT1, exploring its substrate-binding kinetics and catalytic mechanisms through various biochemical approaches.^42^^,^^43^ Our kinetic analysis confirmed moderately enhanced catalytic efficiency of hPRMT1 compared to mPRMT1 (Figure S1E and Table S1). We hypothesize that hPRMT1 maintains a conformation suitable for secondary methylation while allowing substrate rotation after monomethylation, whereas mPRMT1 is expected to dissociate monomethylated substrates faster than hPRMT1 after MMA generation and have a lower efficiency of processive ADMA formation compared to hPRMT1. These findings suggest that the substitution at position 179 (His in mouse, Tyr in human) may have fine-tuned the enzyme’s catalytic behavior, leading to enhanced processivity and/or substrate recognition capacity in hPRMT1. To further support this notion, ProteinMPNN,^27^ a state-of-the-art protein design algorithm, consistently predicted tyrosine over histidine at position 179 (Figure 3D). One plausible mechanistic rationale for MPNN’s preference is that, Y179 in hPRMT1 establishes stabilizing interactions with residues near the SAM-binding site, such as E147, T176, D182, and K183, significantly enhancing local structural integrity compared to H179 in mPRMT1 (Figure S2A). The presence of this mutation specifically in human PRMT1 among mammals raises an interesting question about the potential functional implications of this amino acid change.

In terms of our focus on this substitution at histidine-179 in mouse PRMT1 to tyrosine in human PRMT1 accompanied by the enhanced arginine methylation activity, the genetic codon of histidine (CAC/CAT) and tyrosine (TAC/TAT) differs by a single nucleotide underlined. This point substitution is relatively common and sometimes it can change protein functions. For instance, the H1047Y mutation in phosphatidylinositol 4,5-bisphosphate 3-kinase catalytic subunit alpha isoform (PIK3CA) enhances its catalytic function, resulting in increased phosphorylation of the Akt kinase and activation of downstream signaling pathways critical for cell survival and proliferation.^44^ Also, in lysosomal acid lipase (LAL), the H274Y mutation causes a dramatic reduction in LAL activity, resulting in cholesteryl ester storage disease.^45^ A possible cause of this change from histidine to tyrosine in protein function would be as follows. One is the different chemical properties of the two amino acids. Histidine contains an imidazole side chain with a p*K*a around 6.0, allowing it to act as both a proton donor and acceptor near physiological pH, and this flexibility is crucial in enzyme active sites, but may introduce variability in binding interactions. In contrast, tyrosine has a phenolic side chain with a higher p*K*a (∼10.5), rendering it less prone to protonation under physiological conditions. Second, the aromatic ring of tyrosine can engage in π-π stacking and hydrogen bonding, potentially enhancing the stability of the substrate-binding interactions. Third, even if not directly in the active site, the histidine to tyrosine change of PRMT1 could alter the reactivity of enzyme-substrates or its dynamics in a way that indirectly affects its catalytic activity (Figures 2 and 3). Indeed, the distances between the methyl group of SAM were shown to confirm a closer and more stable interaction between human PRMT1 and the substrate compared to mouse PRMT1. This could be through long-range interactions or by influencing the PRMT1’s ability to change reactivity with substrates. In addition to the view of the above, when such an amino acid substitution occurs at a critical histidine residue, it can change protein functions, and this notion suggests the potential importance of re-evaluating the impact of apparently neutral amino acid substitutions in functional studies of proteins.

One of the most striking findings of this study was the age-related differences in response to LPS-induced inflammatory stress; that is, lower TNF-α levels of huMice than WT mice at 12-month-old (Figure 6A). This observation appears to be consistent with the concept of “inflammaging”, which refers to the chronic, low-grade inflammation that develops during aging.^46^ In general, inflammaging is characterized by increased levels of pro-inflammatory cytokines, e.g., TNF-α and IL-6, and is closely associated with cellular senescence and inflammatory dysregulation.^47^ It should be noted that inflammaging is known to be a major contributor to the development of age-related diseases such as cardiovascular disease, neurodegenerative disorders, and metabolic syndrome, where persistent inflammation activation intensifies tissue damage over time.^47^^,^^48^ In this regard, the reduced inflammatory response observed in 12-month-old huMice suggests that enhanced catalytic activity of PRMT1 could play a protective role in mitigating the effects of inflammaging, thus conferring resistance to age-associated inflammatory stress (Figure 6). Thus, huMice will serve as a valuable model for understanding the physiological significance of arginine methylation and also allow us to find new therapeutic strategies for human diseases where stress and inflammatory responses are critical factors.

By examining transcriptional networks at multiple ages and in distinct tissues, we demonstrated that hPRMT1 consistently aligns with biological pathways governing inflammatory and stress responses, revealing its capacity to shape organism-wide regulatory circuits. To probe these insights further, we defined a novel hPRMT1-associated inflammatory response network that integrates immune regulation and stress adaptation genes into a coherent framework of functional interconnections. Importantly, this network becomes more active in huMice under LPS-induced inflammatory stress, confirming its physiological relevance and reinforcing the concept that hPRMT1 extends beyond basic metabolic roles. The observed network expansion suggests that hPRMT1 might have broader substrate specificity, potentially enabling it to regulate additional physiological processes beyond fundamental metabolic functions (Figures 7A and 7B). Furthermore, the hPRMT1 network serves as a valuable resource for connecting PRMT1 activity to inflammation-related diseases and exploring their molecular foundations.

Notably, our detailed biochemical assays further support this notion of substrate-dependent catalytic enhancement. While canonical PRMT1 substrates such as histone H4 and EWS exhibited significantly enhanced methylation by hPRMT1, no such catalytic advantage was observed for the non-RGG substrate FOXO1 (Figure S1F). This substrate-selective behavior implies that the structural and dynamic consequences of the H179Y substitution preferentially enhance interactions with specific arginine-containing motifs, rather than indiscriminately increasing PRMT1’s overall catalytic efficiency. Future proteomic profiling will be essential for comprehensively delineating the scope and limits of this substrate specificity.

Taken together, these findings exemplify how the thoughtful integration of molecular, computational, and experimental approaches can illuminate the complex, systemic effects of a single amino acid change, ultimately linking subtle molecular alterations to pronounced physiological resilience.

### Limitations of the study

While our RNA-seq analyses suggest that huMice exhibit anti-aging effects at the transcriptome level, further experimental validation is necessary to determine the extent to which these changes translate into physiological or functional outcomes. Additionally, although huMice displayed increased resistance to LPS-induced inflammatory stress, it remains unclear whether similar resistance extends to other types of physiological or environmental stressors. Future studies are needed to assess the generalizability of the anti-stress phenotype and to identify the molecular pathways and substrates involved.

## Resource availability

### Lead contact

Further information and requests for resources and reagents should be directed to and will be fulfilled by the Lead Contact, Akiyoshi Fukamizu (akif@tara.tsukuba.ac.jp).

### Materials availability

This study did not generate new unique reagents. All materials used are commercially available or previously reported.

### Data and code availability

• The RNA-seq datasets generated in this study have been deposited in the DDBJ BioProject repository under the following accession numbers: PRJDB19193 (male MEFs), PRJDB19544 (heart and kidney samples from 2-, 12-, and 21–22-month-old male mice), and PRJDB19610 (heart and kidney samples from 12-month-old male mice treated with LPS). The data are publicly accessible at DDBJ.
• No custom code was generated or used in this study.
• Any additional information required to reanalyze the data reported in this paper is available from the lead contact upon request.

## Acknowledgments

We thank Rie Sato for her assistance in mass spectrometer analysis. We thank the members of Fukamizu laboratory for their helpful discussion. Graphical abstract was created with BioRender.com. The computation was performed using the Pegasus system by Multidisciplinary Cooperative Research Program in Center for Computational Sciences (CCS), Univerisyt of Tsukuba and partially supported by Center for Quantum Information Life Sciences (QILS), Univerisyt of Tsukuba. This work was supported by the 10.13039/501100001691Japan Society for the Promotion of Science Grant-in-aid for Scientific Research (A) (23H00321 to A.F.) and a grant from AMED-CREST, AMED (JPgm1410010 to A.F.).

## Author contributions

Y.Y., K.M., and A.F. designed the study. Y.Y., K.M., and H.M. conducted mouse experiments including MEF-related cultures. Y.Y., K.M., M.M., and J.-D.K. performed transcriptome analyses. Y.Y., K.K., S.M., F.K., S.T.-F., and H.D. carried out biochemical and mass spectrometric analyses. K.H. and Y.S. performed molecular dynamics simulations and trajectory analysis. Y.Y., K.M., H.D., J.-D.K., and A.F. wrote the manuscript.

## Declaration of interests

The authors declare no competing interests.

## STAR★Methods

### Key resources table

**Table:** 

REAGENT or RESOURCE	SOURCE	IDENTIFIER
**Antibodies**		

Anti-p21antibody [EPR18021]	Abcam	Cat# ab188224; RRID: AB_2734729
Goat Anti-Rabbit IgG H&L (Alexa Fluor® 555)	Abcam	Cat# ab150078; RRID: AB_2722519

**Bacterial and virus strains**		

Escherichia coli BL21-CodonPlus (DE3)-RIL	Agilent	230240

**Biological samples**		

Mouse tissues (heart, kidney, spleen, etc.)	This paper	N/A
Mouse embryonic fibroblasts (MEFs)	This paper	N/A

**Chemicals, peptides, and recombinant proteins**		

Adenosyl-L-methionine, S-[methyl-^3^H]-	Revvity	NET155V
S-(5′-Adenosyl)-L-methionine chloride dihydrochloride	Sigma-Aldrich	A7007
*N*^*G*^-Monomethyl-L-arginine, acetate	Dojindo	Cat#*N*-4111; CAS: 53308-83-1 (free base)
*N*^*G*^*,N*^*G*^-Dimethylarginine dihydrochloride	Sigma Aldrich	Cat#D4268; CAS: 220805-22-1 (free base)
Formic Acid (abt. 99%)	FUJIFILM Wako Pure Chemical Corporation	Cat#067-04531; CAS: 64-18-6
*N*^w^-Propyl-L-Arginine hydrochloride	Sigma Aldrich	Cat#SML2341; CAS:137361-05-8 (free base)
Distilled Water	FUJIFILM Wako Pure Chemical Corporation	Cat#042-16973; CAS: 7732-18-5
Acetonitrile	FUJIFILM Wako Pure Chemical Corporation	Cat#015-086331; CAS: 75-05-8
6 mol/L Hydrochloric Acid	FUJIFILM Wako Pure Chemical Corporation	Cat#082-05421; CAS: 7647-01-0
Synthetic EWS-RGG3 peptide: (NH_2_-GRGGPGGMRGGRGGLMDR GGPGG-MFRGGRGGDRGG-COOH)	Sigma-Aldrich Japan	https://www.merckgroup.com/jp-ja/company/sigma-aldrich.html
Histone H4 Human, Recombinant	New England Biolabs	M2504
GST-tagged hPRMT1 protein	This paper	N/A
GST-tagged mPRMT1 protein	This paper	N/A
PreScission Protease	Cytiva	27-0843-01
Glutathione Magnetic Agarose	Pierce	78602
PrimeSTAR® Max DNA Polymerase	Takara Bio	R045A
*Dpn* I	Takara Bio	1235A
LB Broth, Miller	Nacalai Tesque	20068-04
Ampicillin Sodium Salt, Animal-Free	Nacalai Tesque	19769-22
Isopropyl-β-D-thiogalactopyranoside, Dioxane free	Nacalai Tesque	19742-94
HEPES	Nacalai Tesque	17514-15
10 mol/L-Sodium Hydroxide Solution	Nacalai Tesque	94611-45
Sodium Chloride	Nacalai Tesque	31319-45
2-Mercaptoethanol	FUJIFILM Wako Pure Chemical Corporation	133-14571
Phenylmethylsulfonyl Fluoride	Nacalai Tesque	27327-52
Potassium Dihydrogenphosphate	Nacalai Tesque	28721-55
Disodium Hydrogenphosphate	FUJIFILM Wako Pure Chemical Corporation	197-02865
Hydrochloric Acid	FUJIFILM Wako Pure Chemical Corporation	080-01066
Dithiothreitol	Nacalai Tesque	14112-52
Imidazole	Nacalai Tesque	19004-35
Glycerol	Nacalai Tesque	17017-93
Tris(hydroxymethyl)aminomethane	Nacalai Tesque	02435-15
DAPI solution	Nacalai Tesque	19178-91
BODIPY™ 493/503 (4,4-Difluoro-1,3,5,7,8-Pentamethyl-4-Bora-3a,4a-Diaza-*s*-Indacene)	Thermo Fisher Scientific	D3922
Lipopolysaccharides from Escherichia coli O55:B5	Sigma-Aldrich	L2880-100MG
LPS-EB (Standard LPS from E.coli 0111:B4)	INVIVOGEN	14874-24
Recombinant Mouse TNF-alpha (aa 80–235) Protein	R&D systems	410-MT-010/CF
TB Green® Premix Ex Taq™ II (Tli RNaseH Plus), Bulk	Takara Bio	RR820L
4%-Paraformaldehyde Phosphate Buffer Solution	Nacalai Tesque	09154-85
DMEM (4.5g/L Glucose) with L-Gln and Sodium Pyruvate, liquid	Nacalai Tesque	08458-16
2.5g/L-Trypsin/1mmol/L-EDTA Solution, with Phenol Red	Nacalai Tesque	32777-44
MEM Non-Essential Amino Acids Solution (100X)	Thermo Fisher Scientific	11140-050
Gentamicin Sulfate Solution(10mg/ml)	Nacalai Tesque	16672-04
ISOGEN II	NIPPON GENE	311-07361
Ethanol (99.5)	FUJIFILM Wako Pure Chemical Corporation	057-00456
PrimeScript™ RT reagent Kit with gDNA Eraser (Perfect Real Time)	Takara Bio	RR047B

**Critical commercial assays**		

BCA Protein Assay Kit	Nacalai Tesque	06385-00
Wizard® *Plus* SV Minipreps DNA Purification Systems	Promega	A1460
Wizard® SV Gel and PCR Clean-Up System	Promega	A9282
LBIS Mouse TNF-α ELISA Kit	FUJIFILM Wako Pure Chemical Corporation	634-44721
Viability/Cytotoxicity Multiplex Assay Kit	DOJINDO	CK17
Senescence β-Galactosidase Staining Kit	Cell Signaling Technology, Inc.	9860S

**Deposited data**		

RNA-seq raw data	This paper	PRJDB19193, PRJDB19544, PRJDB19610
Normalized RNA-seq count data from multiple organs and ages (used for GSEA)	Schaum et al.^32^	https://www.ncbi.nlm.nih.gov/geo/query/acc.cgi?acc=GSE132040
Mouse reference genome (GRCm39/mm39)	Genome Reference Consortium	https://www.gencodegenes.org/mouse/

**Experimental models: Organisms/strains**		

Mouse: C57BL/6J mice	CLEA Japan Inc.	N/A
Mouse: humanized PRMT1 knock-in mice (H179Y)	This paper	N/A

**Oligonucleotides**		

CRISPR sgRNA targeting Prmt1 (20-mer: CAACACACCGTGCTGC ACG-CTC)	This paper	N/A
ssODN donor for knock-in (see method details for full sequence)	This paper	N/A
RT-qPCR primers, see Table 1	This paper	N/A
Primer: N-terminal deleted mPRMT1 Forward: ATGCTCAACACCGTGCT CCATGCCCGGGACAAGTGGCTG	This paper	N/A
Primer: N-terminal deleted mPRMT1 Reverse: CAGCCACTTGTCCCGGG CATGGAGCACGGTGTTGAGCAT	This paper	N/A
Primer: mFOXO(37-338) Forward: CGGATCCTCGACCACCTCCAG TCCGGCGCCGTC	This paper	N/A
Primer: mFOXO(37–338) Reverse: GCCTATTACCCATCTCCCAGGTC ATCCTGCTCT	This paper	N/A

**Recombinant DNA**		

Plasmid: full-length hPRMT1	This paper	N/A
Plasmid: full-length mPRMT1	This paper	N/A
Plasmid: N-terminal deleted hPRMT1	Toma-Fukai et al.^49^	N/A
Plasmid: N-terminal deleted mPRMT1	This paper	N/A
Plasmid: mFOXO (37–338)	This paper	N/A

**Software and algorithms**		

UCSF Chimera v1.17.1	UCSF Resource for Biocomputing, Visualization, and Informatics	https://www.cgl.ucsf.edu/chimera/
Modeller (via Chimera)	Andrej Sali Lab, UCSF	https://salilab.org/modeller/
Amber 22	Amber Developers (University of California & collaborators)	https://ambermd.org/
Gaussian 16, GaussView 6.0	Gaussian, Inc.	https://gaussian.com
Trim Galore v0.6.10	Babraham Institute (Bioinformatics Group)	https://www.bioinformatics.babraham.ac.uk/projects/trim_galore/
FastQC v0.11.9	Babraham Institute (Bioinformatics Group)	https://www.bioinformatics.babraham.ac.uk/projects/fastqc/
STAR v2.7.10a	Alexander Dobin (Cold Spring Harbor Lab)	https://github.com/alexdobin/STAR
featureCounts v2.0.3	Subread Team, The Walter and Eliza Hall Institute	https://subread.sourceforge.net/featureCounts.html
DESeq2	Love M et al.^50^	https://bioconductor.org/packages/release/bioc/html/DESeq2.html
clusterProfiler	Yu G et al.^51^	https://bioconductor.org/packages/clusterProfiler/
WGCNA	Langfelder P et al.^41^	https://cran.r-project.org/web/packages/WGCNA/index.html
ComBat (sva package)	Bioconductor	https://bioconductor.org/packages/release/bioc/html/sva.html
STRING	Swiss Institute of Bioinformatics	https://string-db.org/
GSEA v4.3.2	Broad Institute	https://www.gsea-msigdb.org/gsea/index.jsp
ProteinMPNN	Dauparas J et al.^27^	https://github.com/dauparas/ProteinMPNN
GraphPad Prism 10	MDF	https://www.graphpad.com/
Cas-OFFinder	Seoul National University	http://www.rgenome.net/cas-offinder/
RING 4.0	BioComputingUP, University of Padua	https://ring.biocomputingup.it/
Biorender	BioRender	https://www.biorender.com
R version 4.3.1	The R Project for Statistical Computing	https://www.r-project.org/
Cytoscape	Otasek et al.^52^	https://cytoscape.org/
LabSolutions LCGC 5.53 sp2	Shimadzu Corporation	https://www.shimadzu.com/

**Other**		

Multi-Beads Shocker	YASUI KIKAI	MB601U
SeQuant ZIC-HILIC column (2.1 × 150 mm)	Merck KGaA	Cat#1.50434.0001
SeQuant ZIC-HILIC guard fitting (1.0 × 14 mm)	Merck KGaA	Cat#1.50448.0001
Ni Sepharose 6 Fast Flow	Cytiva	17531802
HiTrap™ Q HP	Cytiva	17115401
HiTrap™ SP HP	Cytiva	17115201
Poly-Lysine coated-glass-bottomed dishes	Matsunami	D11131H

### Experimental model and study participant details

#### Animal studies

All animal husbandry and experiments were approved by the Institutional Animal Experiment Committee of the University of Tsukuba and conducted in accordance with the Fundamental Guidelines for Proper Conduct of Animal Experiments and Related Activities in Academic Research Institutions under the jurisdiction of the Ministry of Education, Culture, Sports, Science, and Technology of Japan. All experiments adhered to the ARRIVE guidelines. Animals were housed in a pathogen-free barrier facility with a 12-h light/dark cycle and provided *ad libitum* access to standard chow (MF diet, ORIENTAL YEAST Co., Ltd, Tokyo, Japan) and water. Age- and sex-matched littermates were used for all experiments. Sex-specific analyses were not performed. Specific ages used for each experiment are described in the corresponding sections of the method details.

#### Humanized PRMT1 mice (huMice)

C57BL/6 male and female mice were obtained from CLEA Japan (Tokyo, Japan). To generate huMice, the CRISPR/Cas9 system was used to introduce a single amino acid substitution at position 179 (His->Tyr) in the mouse *Prmt1* gene. A 20-mer oligonucleotide (CAACACACCGTGCTGCACG-CTC) adjacent to the PAM site was selected as a target sequence for sgRNA design, and the sgRNA was co-expressed with Cas9 protein using the PX330 vector. To carry out homology-directed repair, a single-stranded donor oligonucleotide containing the above amino acid substitution site and homology arm flanking the mutation site were synthesized. The donor sequence used was: 5′-ATGGGTTACTGCCTCTTCTACGAGTCCATGCTCAACACCGTGCT-GTACGCTCGGGACAAGTGGCTGGTGAGCCATACGCCTTGGATGGGCG-3’.

The CRISPR/Cas9 complex, along with the donor template, was microinjected into fertilized C57BL/6J zygotes. To evaluate the specificity of the sgRNA and minimize potential off-target effects, in silico genome-wide off-target prediction was conducted using Cas-OFFinder^53^ against the GRCm38/mm10 mouse genome. The 20-nt guide sequence and NGG PAM were used, and no potential off-target sites with zero mismatches or bulges were identified outside the intended locus on chromosome 7. A relaxed search using a truncated 15-mer version of the guide RNA also failed to detect additional off-target candidates. Furthermore, the founder mice were backcrossed with C57BL/6J mice for three generations to dilute potential off-target events. To confirm the conversion of the mouse Prmt1 allele to humanized allele and the precise incorporation of the H179Y mutation, DNA sequencing was performed using a 3130xl Genetic Analyzer (Applied Biosystems).

#### Mouse embryonic fibroblasts

Mouse embryonic fibroblasts (MEFs) were isolated from E12.5 male embryos of WT mice and huMice following the RIKEN protocol. After removing the head, tail, limbs, and internal organs, embryos were minced in ice-cold PBS without calcium and magnesium (PBS(−)) to remove blood from the tissue. The PBS(−) was then removed using a pipette, and the minced embryos were digested with 2.5 g/L Trypsin/1 mmol/L EDTA solution (Nacalai Tesque, Inc., Kyoto, Japan) at 37°C for 15 min. The digested embryos were dispersed by pipetting, and an equal volume of fetal bovine serum (FBS) was added to stop the digestion. The resulting suspension was filtered through a 100 μm cell strainer and maintained in Dulbecco’s Modified Eagle Medium (DMEM; Nacalai Tesque, Inc., Kyoto, Japan) supplemented with 10% FBS (Thermo Fisher Scientific, Waltham, MA, USA), 1% penicillin/streptomycin (Nacalai Tesque, Inc., Kyoto, Japan), and 1% MEM Non-Essential Amino Acids (Thermo Fisher Scientific, Waltham, MA, USA). Cell suspensions from individual embryos were plated separately into 100-mm diameter culture dishes and cultured at 37°C in a humidified incubator with 5% CO_2_. All MEFs were routinely tested for mycoplasma contamination and consistently found to be negative.

### Method details

#### Molecular dynamics simulation

In this study, we focus on the human (h) and mouse (m) dimeric PRMT1-RGG-SAM complexes. The dimeric PRMT1 obtained from the Protein DataBank with PDB ID 6NT2^54^ was considered as hPRMT1. For the mPRMT1, tyrosine at position 179th of hPRMT1 was mutated to histidine using UCSF Chimera 1.17.1.^55^ To generate the complex structures of these PRMT1 and RGG substrates, the coordinates of an RGG moiety were superimposed with that in the monomeric PRMT1-RGG-SAM complexes (PDB ID: 1OR8). The missing amino acid residues of the RGG substrate with 35 amino acids (GRGGPGG MRGGRGGLMD RGGPGGMFRG GRGGDRGG) was subsequently added using homology modeling techniques by Modeller software via UCSF Chimera 1.17.1.^55^ Then, the 3D coordinates of RGG and SAM were transferred to the dimeric h and mPRMT1s.

The dimeric PRMT1-RGG-SAM complexes were assigned for the protonation state at neutral pH using the PDB2PQR web server.^56^ The force field parameter and topology files for SAM were prepared using gaussview 6.0 and Gaussian16^57^ at the DFT-B3LYP/6-31G∗ basis set of theory and Antechamber based on the General Amber Force Field 2 (GAFF2),^58^ as previously described.^59^^,^^60^^,^^61^ These complexes were solvated by TIP3P water molecules into a cubic box with a 13 Å minimum distance between the protein surface and the edge of the solvent box and neutralized by 8 Na+ ions. The ff19SB force field^62^ was employed for amino acids.

The MD simulations were performed using the AMBER 22 package program^63^ in the NVT ensemble. After the system was equilibrated, MD simulations in the NPT ensemble were further conducted. In the MD simulation, a time step of 2 fs was used, the periodic boundary condition was adopted, and the bonds involving hydrogen atoms were constrained by SHAKE, a widely accepted method in the MD simulations. Electrostatic energy was calculated using the Particle Mesh Ewald (PME) method, with a non-bonded cutoff of 12.0 Å. These MD simulations were heated up to 310 K for 1 ns, and then the temperature during the MD simulation was maintained at 310 K by the Langevin temperature equilibration scheme, with a coupling time of 2 ps, for 300 ns with three replicates for each hPRMT1 and mPRMT1. The MD trajectory was analyzed using the CPPTRAJ module^64^ implemented in AmberTools 21,^65^ evaluating the systems in terms of distance stability between a key arginine in the RGG substrate and the methyl group in SAM and hydrogen bonding analysis. Additionally, the dihedral angles of the amino acid at position 179th in both systems were measured to distinguish the conformational differences of the tyrosine and histidine side chains in hPRMT1 and mPRMT1. Note that the protonation state for H179 was assigned as HID (neutral, δ-protonated), as predicted by PROPKA3.0 via PDB2PQR.

The residue network interactions for hPRMT1 and mPRMT1 were constructed using the Residue Interaction Network Generator^66^ and visualized by Cytoscape 3.10.1.^52^

#### Protein purification and *in vitro* methylation assay

Full-length GST-tagged hPRMT1 and mPRMT1 were expressed in *Escherichia coli* using the pGEX-6P-1 constructs. The constructs were transformed into chemically competent BL21-CodonPlus (DE3)-RIL cells (*Agilent Technologies*). Protein expression was induced by adding 0.5 mM IPTG and incubating for 4 h at 22°C. Recombinant GST-tagged proteins were purified using Pierce Glutathione Magnetic Agarose Beads (*Thermo Fisher*), and the GST tag was cleaved by incubating with PreScission Protease (Cytiva) for 4 h at 4°C.

N-terminal 44 amino acids deleted hPRMT1 and mPRMT1 were expressed using the pET44a vector.^49^ mPRMT1 vector was prepared with a single mutation using hPRMT1 vector as the template. mFOXO1(37–338), containing the 37–338 amino acids, was expressed using the pMAL-c2X vector gifted from Dr. Yoshizawa of Ritsumeikan University. The constructs were transformed into chemically competent Rosetta2(DE3) (Merck). Protein expression was induced by adding 0.1 mM IPTG and incubating overnight at 18°C. Recombinant His-tagged proteins were purified using Ni Sepharose Beads (Cytiva), and tags were cleaved by PreScission Protease overnight at 4°C. Tag removal PRMT proteins and mFOXO(37–338) were further purified using HitrapQ HP and Hitrap SP(Cytiva), respectively.

*In vitro* methylation assays were performed with GST-hPRMT1 or GST-mPRMT1 (0.5 μg) together with recombinant histone H4 (2 μg) and GST-RGG3 (1 μg) in the presence of 1 μCi [^3^H]-SAM (*PerkinElmer*) as the methyl donor. After incubation at 30°C for 40 min, the reaction products were separated on SDS-PAGE and gels were stained with Coomassie blue, followed by soaking in Amplify fluorographic reagent (GE Healthcare) for 20 min. The gels were dried under vacuum at 80°C for 1h and then exposed to Amersham Hyperfilm ECL (GE Healthcare) to visualize the methylation signals.

#### Time-course quantitative methylation assay

To compare the specific activity of hPRMT1 and mPRMT1, a synthetic peptide corresponding to positions 564–596 of human EWS-RGG3 (GRGGPGGMRGGRGGLMDRGGPGGMFRGGRGGDR, Sigma-Aldrich Japan) and recombinant human histone H4 (New England Biolab) were used as substrates for the methylation assay. Notably, the peptide used in this methylation assay is identical in sequence to the one employed in our molecular dynamics simulation (MDS), allowing for a direct comparison between the computational and experimental results. Substrates (132.7 pmol of EWS peptide and 1.5 μg of histone H4) were incubated with 0.5 μg of recombinant hPRMT1 or mPRMT1 and 10 nmol of SAM in a final volume of 50 μL at 30°C for various times (2.5, 5, 10, 20, 40 h). Methylation reactions were stopped by snap freezing and stored at −80°C and concentrated with centrifugal evaporator (EYELA) before acid hydrolysis.

#### PRMT1 Midchaelis-Menten enzyme kinetics

Steady-state kinetic analyses were performed using various concentrations of RGG peptide as substrate. The reactions were initiated by adding 200 nM of purified hPRMT1 or mPRMT1 to a mixture containing 50 μM S-adenosylmethionine (SAM) and the RGG peptide. The reactions were incubated at 30°C for 120 min and quenched by the addition of 0.1% trifluoroacetic acid (TFA). Reaction products, specifically monomethylarginine (MMA) and asymmetric dimethylarginine (ADMA), were quantified by LC-MS/MS using external standard calibration curves.

For MMA analysis, substrate concentrations ranged from 0 to 100 μM. For ADMA, concentrations were limited to 0 to 5 μM due to product formation saturation at higher substrate levels.

Calculation of Initial reaction velocities and kinetic parameters product concentrations (in μM) were converted to initial reaction velocities (v) in nM/min as follows:

$$v=\frac{\left[\text{Product}\right]\times 1000}{t}$$

Where [Product] is the measured concentration in μM and t = 120 min is the reaction time. Velocities were plotted against the corresponding substrate concentrations and fitted to the Michaelis-Menten equation using nonlinear regression in GraphPad Prism 10:

$$v=\frac{V_{\max}\cdot \left[S\right]}{K_{m}+\left[S\right]}$$

Kinetic parameters and were extracted from the fitted curves. Catalytic turnover rate and catalytic efficiency were then calculated using the enzyme concentration of 200 nM:

$$k_{\text{cat}}=\frac{V_{\max}}{\left[E\right]}$$

#### FOXO1 methylation

To evaluate PRMT1 substrate specificity beyond RGG motifs, *in vitro* methylation assays were conducted using recombinant mouse FOXO1 (mFOXO1, amino acids 37–338) as substrate. The reaction mixture contained 2.66 μM FOXO1, 0.1 μg/μL hPRMT1 or mPRMT1, and 50 μM SAM in reaction buffer. Reactions were carried out at 30°C for 6 h.

After incubation, reaction products were immediately subjected to SDS-PAGE. The gels were stained using a negative staining protocol with EzStainReverse (ATTO, AE-1310). Gel bands corresponding to FOXO1 were excised, crushed into gel microparticles (gel slurry), and filtered through ATTO Prep MF cartridges (ATTO, 3521370). Proteins were extracted from the eluate via methanol–chloroform extraction. The purified FOXO1 proteins were hydrolyzed and subsequently analyzed by LC-MS/MS to quantify arginine methylation levels.

#### LC-MS/MS analyses

All samples were dissolved in 6N HCl, and hydrolysis was carried out by heating at 110°C for 24 h after adding 115 pmol of *N*-propyl-L-arginine (*N*-PLA) as an internal standard. The excessive HCl was removed by dry distillation from the hydrolysate with the centrifugal evaporator, then redissolved in 50 μL of purified water. Each methylarginine from protein/peptide hydrolysates was quantified using an LCMS-8050 triple quadrupole mass spectrometer (Shimadzu, Kyoto, Japan) connected to a Nexera ultra high-pressure liquid chromatography system (Shimadzu). Individual analytes were separated on a SeQuant ZIC-HILIC column (2.1 × 150 mm, Merck KGaA, Darmstadt, Germany) fitted with a SeQuant ZIC-HILIC guard fitting (1.0 × 14 mm, Merck KGaA) as previously described,^67^ with modifications to the LC elution conditions and multiple reaction monitoring (MRM) transitions as detailed below. Samples (10 μL) dissolved in water were injected at a flow rate of 0.2 mL/min and mobile phases A and B were prepared with a ratio of water: acetonitrile: formic acid of 98:1:1 and 1:98:1, respectively, and the gradient elution program as follows: 95% B for 1 min, reduced to 5% over 9 min, maintained at 5% for 4 min, equilibrated at 95% B for 7 min until next analysis. The multiple reaction monitoring transitions of arginine, MMA, ADMA, SDMA and N-PLA were used *m/z* 175 > 70.05, 189.15 > 70.15, 203.15 > 158.05, 203.15 > 171.95 and 216.60 > 70.15, respectively.

System operation and data acquisition and processing were completed with LabSolutions for LC-MS Ver. 5.60 software (Shimadzu). Based on the data obtained from the standards, calibration curves for the measurement of arginine, methylarginines, and N-PLA in the range of 0.1–10 pmol were constructed. Measured concentrations of the arginine derivatives in each sample were calculated from the calibration curves.

#### Mice sample preparation

Under anesthesia with isoflurane, mice organs were promptly harvested and snap-frozen in liquid nitrogen. To ensure tissue integrity and prevent thawing, frozen organs were clashed into powder using a Multi-Beads Shocker (Yasui Kikai) while maintaining temperatures below freezing throughout the process.

#### Protein extraction

Tissue powders were dissolved in phosphate-buffered saline (PBS (−)) containing a 1× protease inhibitor cocktail (Nacalai Tesque), ensuring the prevention of protease activity during the extraction process. Protein concentrations were subsequently determined using the bicinchoninic acid (BCA) assay (Nacalai Tesque).

#### LPS treatment

Lipopolysaccharide (LPS) from *Escherichia coli* O55 (*Sigma, Rehovot, Israel*) was used to induce an inflammatory response in C57BL/6J WT mice and huMice. Mice were intraperitoneally injected with either 2 mg/kg LPS or saline as a control. For measuring plasma *Tnf* and *Il6* levels, blood and tissues were collected 4 h post-injection. Plasma tumor necrosis factor alpha (TNFα) levels were measured by LBIS Mouse TNF-α ELISA Kit (Fujifilm Wako Shibayagi, Gumma, Japan). For survival analysis, mice received the same LPS dose and were monitored daily for seven days.

#### Cell proliferation and cellular cytotoxicity

Cell proliferation and cellular cytotoxicity were evaluated using the Viability/Cytotoxicity Multiplex Assay Kit (Dojindo, Kumamoto, Japan), following the manufacturer’s protocol. MEFs were seeded at 20,000 cells/well in a 96-well plate. After 24 h, cell proliferation was measured using the water-soluble tetrazolium salt WST-8 according to the manufacturer’s instructions. Cellular cytotoxicity was assessed after treatment with recombinant mouse TNF-alpha (aa 80–235) protein (R&D Systems, Minnesota, USA) for 24 h. The culture medium was replaced with DMEM containing 1% FBS.

#### Immunofluorescence and senescence-associated β-gal staining

Late passage MEFs were plated in poly-L-lysine-coated glass-bottom dishes (Matsunami, Osaka, Japan) at 10,000 cells per well, fixed with 4% paraformaldehyde in phosphate buffer solution for 20 min at room temperature, and permeabilized for 10 min in PBS containing 0.1% Triton X-100. Fixed cells were blocked with 3% BSA in PBS for 1 h at room temperature and then incubated overnight at 4°C with the primary antibody, rabbit anti-p21 (Abcam, Cambridge, UK). Cells were washed twice with PBS, followed by incubation with Alexa Fluor 555-conjugated anti-rabbit secondary antibody (Abcam, Cambridge, UK) for 1 h at room temperature. Nuclei were counterstained with DAPI (Nacalai Tesque, Inc., Kyoto, Japan). For the visualization of lipid droplets, fixed MEFs were stained with 1 μM BODIPY 493/503 (4,4-Difluoro-1,3,5,7,8-pentamethyl-4-bora-3a,4a-diaza-*s*-indacene) (Thermo Fisher Scientific, Waltham, MA, USA). Images were acquired using a BZ-X810 fluorescence microscope (Keyence Corp., Osaka, Japan) and a Thunder confocal microscope (Leica Microsystems, Wetzlar, Germany).

Cell senescence was assessed with the colorimetric Senescence-Associated β-Gal Staining Kit (Cell Signaling Technology, MA, USA), following the manufacturer’s protocol.

#### RNA-sequencing

Total RNA was extracted using ISOGEN II reagent (Nippon Gene, Tokyo, Japan). 100 ng of total RNA was depleted of ribosomal RNA using the NEBNext rRNA Depletion Kit (New England Biolabs, MA, USA) and then amplified cDNA libraries using the NEBNext Ultra II RNA Library Prep Kit for Illumina (New England Biolabs, MA, USA). Size distribution and concentration of amplified cDNA libraries were validated by an Agilent Bioanalyzer (Agilent, CA, USA) and an Agilent High-Sensitivity DNA kit (Agilent). Sequencing was conducted with a NextSeq 500 sequencer (Illumina, CA, USA) following the manufacturer’s instructions. The RNA-seq datasets generated in this study have been deposited in the DDBJ BioProject repository under the following accession numbers: PRJDB19193 (MEF samples), PRJDB19544 (heart and kidney samples from 2-, 12-, and 21-22-month-old mice), and PRJDB19610 (heart and kidney samples from 12-month-old mice treated with LPS). The data are publicly accessible at DDBJ.

#### ProteinMPNN

To predict the impact of the H179Y substitution on PRMT1, we utilized the ProteinMPNN web application (https://huggingface.co/spaces/simonduerr/ProteinMPNN),^27^^,^^68^ a state-of-the-art algorithm for protein sequence design. The backbone structure of mPRMT1 (PDBID: 1OR8) was used as the input. The model was run 15 times independently to ensure robust prediction results. For each iteration, the algorithm generated amino acid sequences optimized to fit the input backbone structure, allowing us to assess the predicted residue at position 179. The output sequences were analyzed to determine the frequency of predicted amino acids at this critical position, providing insight into the evolutionary and structural preference of tyrosine over histidine.

#### RNA-seq data analysis

RNA-seq data analysis was conducted on a Linux platform. First, raw reads were trimmed using Trim Galore v0.6.10 (*Babraham Bioinformatics*), which removed both adapter sequences and low-quality base calls (Phred quality score < 25). Quality control checks were performed using FastQC v0.11.9 (*Babraham Bioinformatics*). Next, reads were aligned to the mouse reference genome (GRCm38/mm10) using STAR v2.7.10a.^69^ The output BAM files were sorted by coordinates and used for downstream analysis.

Counts matrix was generated using featureCounts v2.0.3.^70^ Differential expression analysis of the resulting counts was performed using DESeq2 in R.^50^ PCA plots were generated using the ggplot2 package. Gene Ontology (GO) enrichment analyses were performed using the clusterProfiler package,^51^ and all *p* values were adjusted using the Benjamini–Hochberg correction.

Gene Set Enrichment Analysis (GSEA) was performed using GSEA software v4.3.2.^71^ For the analysis of age-related gene expression, we reanalyzed previous transcriptome data and defined an “age-associated gene set”^32^ in which gene expression was significantly upregulated in 24-month and 27-month-old mice, as compared to 3-month and 6-month-old mice. The heart and kidney tissues were processed separately to evaluate age-related transcriptional changes.

#### Estimation of PRM1 network using WGCNA analysis

Weighted Gene Co-Expression Network Analysis (WGCNA) was performed to identify gene co-expression modules associated with the differences between WT mice and huMice. The dataset consisted of RNA-seq data from heart and kidney tissues collected at 2, 12, and 21–22 months of age. The data were analyzed separately for WT mice and huMice.

RNA-seq data were normalized using VarianceStabilizingTransformation (VST) in the DESeq2 package^50^ to account for variance in expression across genes. ComBat^72^ was applied to correct for batch effects, ensuring that biological differences were preserved while removing technical variability.

The WGCNA package^41^ in R was used for co-expression analysis. The adjacency matrix was calculated, and a Topological Overlap Matrix (TOM) was constructed. Genes were hierarchically clustered using the average linkage method based on the dissimilarity of the TOM. Gene modules were identified by cutting the resulting dendrogram using the dynamic tree cut method, with a minimum module size of 50 genes. Gene significance (GE) and module membership (MM) values were exported for further analysis.

To further investigate the functional relevance of PRMT1, genes within the PRMT1-associated module from the WGCNA analysis were extracted. The STRING database^73^ was then utilized to integrate known protein-protein interaction data. Only high-confidence interactions (interaction score >0.7) were considered for network expansion. The WGCNA network was merged with the STRING data, creating a combined network for further functional analysis.

Gene Ontology (GO) enrichment analysis was performed on the identified PRMT1 sub-network using STRING functional enrichment^73^ to assign GO terms to each gene based on biological processes. The PRMT1 sub-network was visualized based on GO term associations.

#### Quantitative real-time PCR

One microgram of total RNA was reverse-transcribed into complementary DNA using the PrimeScript RT reagent Kit with gDNA Eraser (TaKaRa, Kyoto, Japan). Quantitative PCR was then performed on a Thermal Cycler Dice Real-Time System (TaKaRa, Kyoto, Japan) using TB Green Premix Ex Taq II (TaKaRa, Kyoto, Japan). Primer sequences used for Tnf, Il6, Prmt1 variants, Cdkn2a (p16), and Cdkn1a (p21) are listed in Table 1. Data were normalized to *Gapdh* using the ΔΔCt method.

### Quantification and statistical analysis

All statistical analyses and graphical representations were performed using GraphPad Prism 10 (GraphPad Software Inc., San Diego, CA, USA). Statistical methods used for each experiment—including the type of test applied, the exact value of n, what n represents (e.g., number of animals, tissue samples, or biological replicates), and the measures of central tendency and variation—are described in the corresponding figure legends (main and supplemental) and/or in the results section. Unless otherwise stated, data are presented as mean ± standard error of the mean (SEM). Statistical significance was assessed using unpaired two-tailed t-test, two-way ANOVA followed by Šídák’s or Tukey’s multiple comparisons test, as appropriate. In all figures, statistical significance is indicated by asterisks as follows: *p* < 0.05 (∗), *p* < 0.01 (∗∗), *p* < 0.001 (∗∗∗), *p* < 0.0001 (∗∗∗). The statistical test used and the meaning of n are provided in each figure legend.

### Additional resources

This work is not part of or involves a clinical trial.
