# Supplementary material for: Protein arginine methylation regulates anti-inflammatory programming in response to aging stress
Source: iScience. 2025 Jul 10;28(8):113095. doi: 10.1016/j.isci.2025.113095 (PMC12312054; doi:10.1016/j.isci.2025.113095)
Supplement: Document S1. Figures S1–S6 and Table S1 [file mmc1.pdf]

iScience, Volume *28*

Supplemental information

Protein arginine methylation regulates anti-inflammatory programming in response to aging stress

Yao Yuan, Kaori Motomura, Jun-Dal Kim, Kowit Hengphasatporn, Koichiro Kako, Syunsuke Maruhashi, Fumiya Kasai, Hayase Mizukami, Sachiko Toma-Fukai, Masafumi Muratani, Yasuteru Shigeta, Hiroaki Daitoku, and Akiyoshi Fukamizu

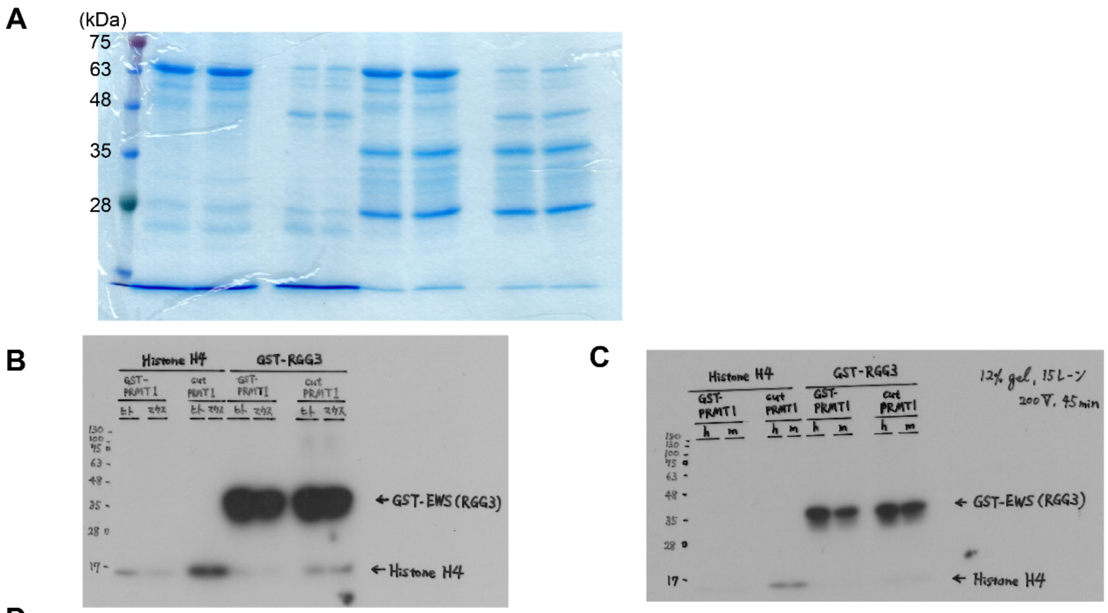

D

N-terminal: GRGGPGGMRGGRGGLMD**R**GGPGGMFRGGRGG-DRGG :C-terminal

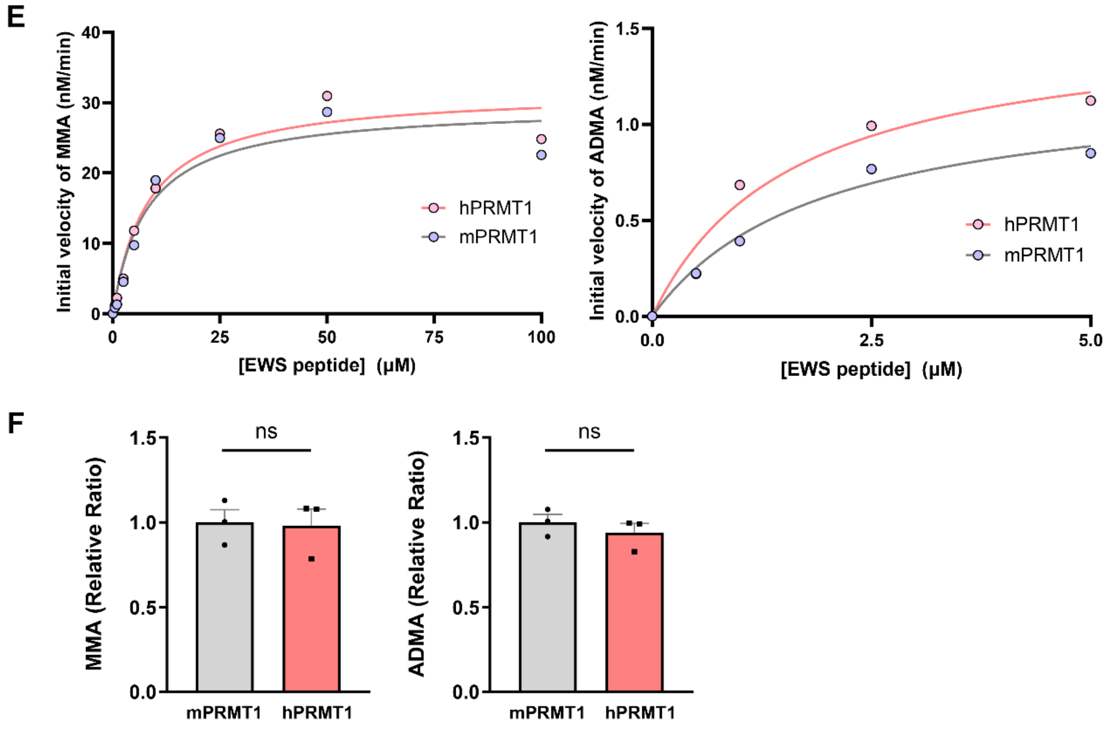

Figure S1. Biochemical characterization of PRMT1 enzymatic activity and substrate methylation *in vitro* (related to Figure 2)

(A) Coomassie Brilliant Blue (CBB) staining of the entire membrane corresponding to the *in vitro* methylation assay shown in Figure 2A–B. This image provides a loading control for all lanes and demonstrates equal protein input. (B) Full autoradiography blot showing methylation of recombinant histone H4. This image corresponds to the data shown in Figure 2A. (C) Full autoradiography blot showing methylation of the EWS-

RGG3 peptide by hPRMT1 and mPRMT1 in the presence of [^{3}H]-SAM. This blot complements the cropped result shown in Figure 2B. (D) Peptide sequences used in the *in vitro* methylation assays and molecular dynamics simulations. The critical arginine residue, which interacts with the SAM methyl group, is highlighted in red. (E) Michaelis-Menten kinetic analysis of MMA and ADMA formation by hPRMT1 and mPRMT1 using the EWS-RGG peptide substrate. Substrate concentrations ranged from 0 to 100 μM for MMA. Due to saturation of ADMA production at high substrate levels, substrate concentrations were limited to 0 to 5 μM. Product formation was measured after 120 minutes and converted to initial velocities (nM/min). All data represent mean values from two independent experiments. (F) Quantification of MMA and ADMA levels in mFOXO1 fragment (amino acids 37–338) following *in vitro* methylation by human PRMT1 (hPRMT1) or mouse PRMT1 (mPRMT1) by LC-MS/MS. MMA and ADMA levels were normalized to mPRMT1 (set to 1.0).Data represent mean ± SD from three independent experiments. *ns,* not significant. Statistical analysis was performed using unpaired two-tailed *t*-test.

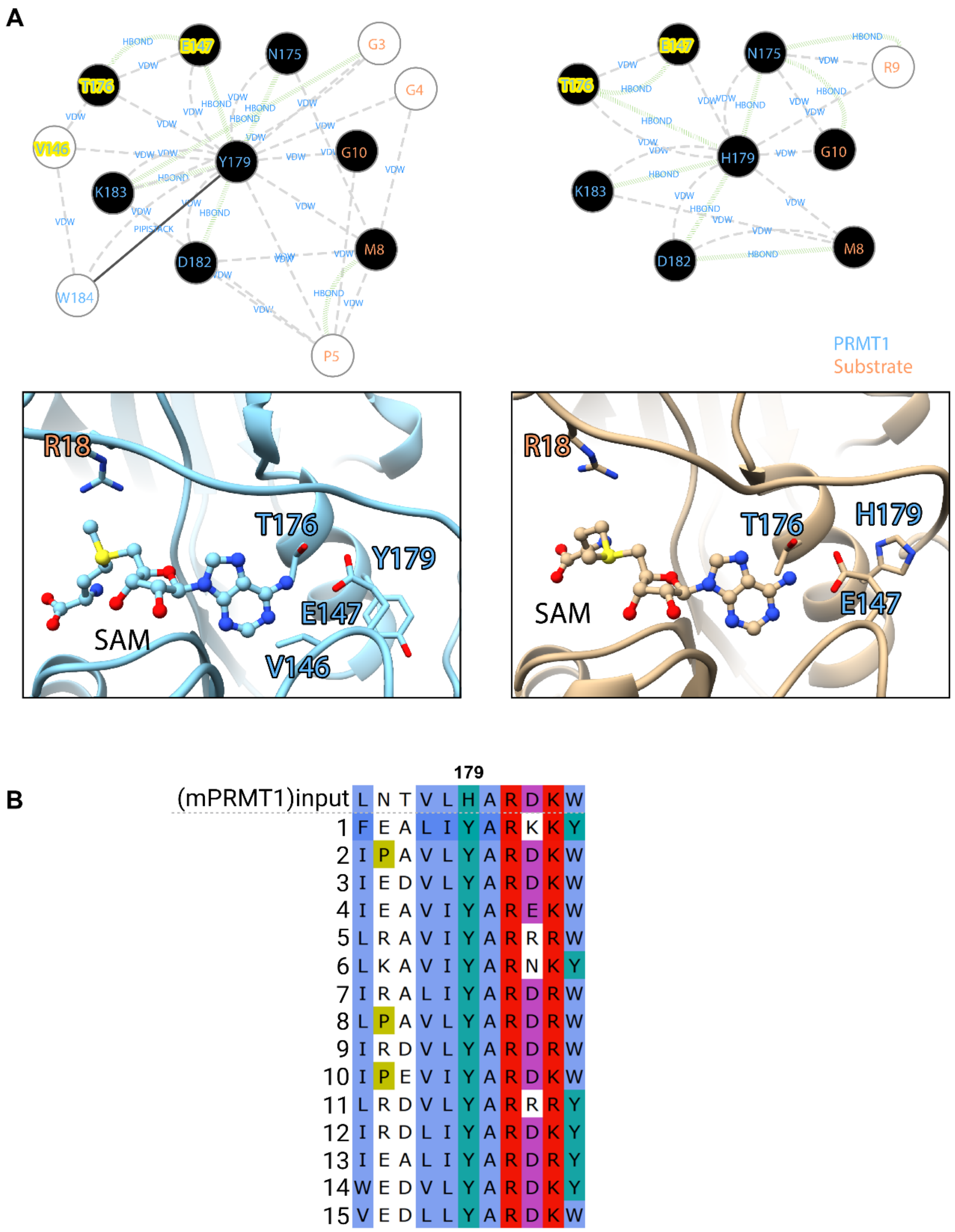

Figure S2. Structural analysis and computational sequence design support Y179 as a stabilizing residue in PRMT1 (related to Figure 3)

(A) (Top) Residue interaction network centered on residue 179 in hPRMT1 (left) and mPRMT1 (right), generated using the Residue Interaction Network Generator (RING) based on MD simulation trajectories. In hPRMT1, Y179 forms multiple stabilizing interactions including hydrogen bonds (HBOND) and van der Waals contacts (VDW), especially with E147, T176, D182, and K183. These interactions contribute to maintaining the structural integrity of the catalytic pocket. In contrast, in mPRMT1,

H179 forms fewer and weaker interactions due to the lack of a hydroxyl group and aromatic π-system. PRMT1 residues are shown in blue, and substrate residues are shown in orange. (Bottom) Representative MD snapshot showing spatial relationships between SAM and surrounding residues in hPRMT1 (left) and mPRMT1 (right), with emphasis on the positioning of residue 179. The structural environment around SAM is more tightly organized in hPRMT1 compared to mPRMT1. (B) ProteinMPNN design results from fifteen independent runs. The top sequence represents the original mPRMT1 amino acid chain, with position 179 and surrounding residues highlighted for clarity. Each of the fifteen generated sequences consistently replaces histidine with tyrosine at position 179, indicating that tyrosine is computationally preferred for structural stability and compatibility.

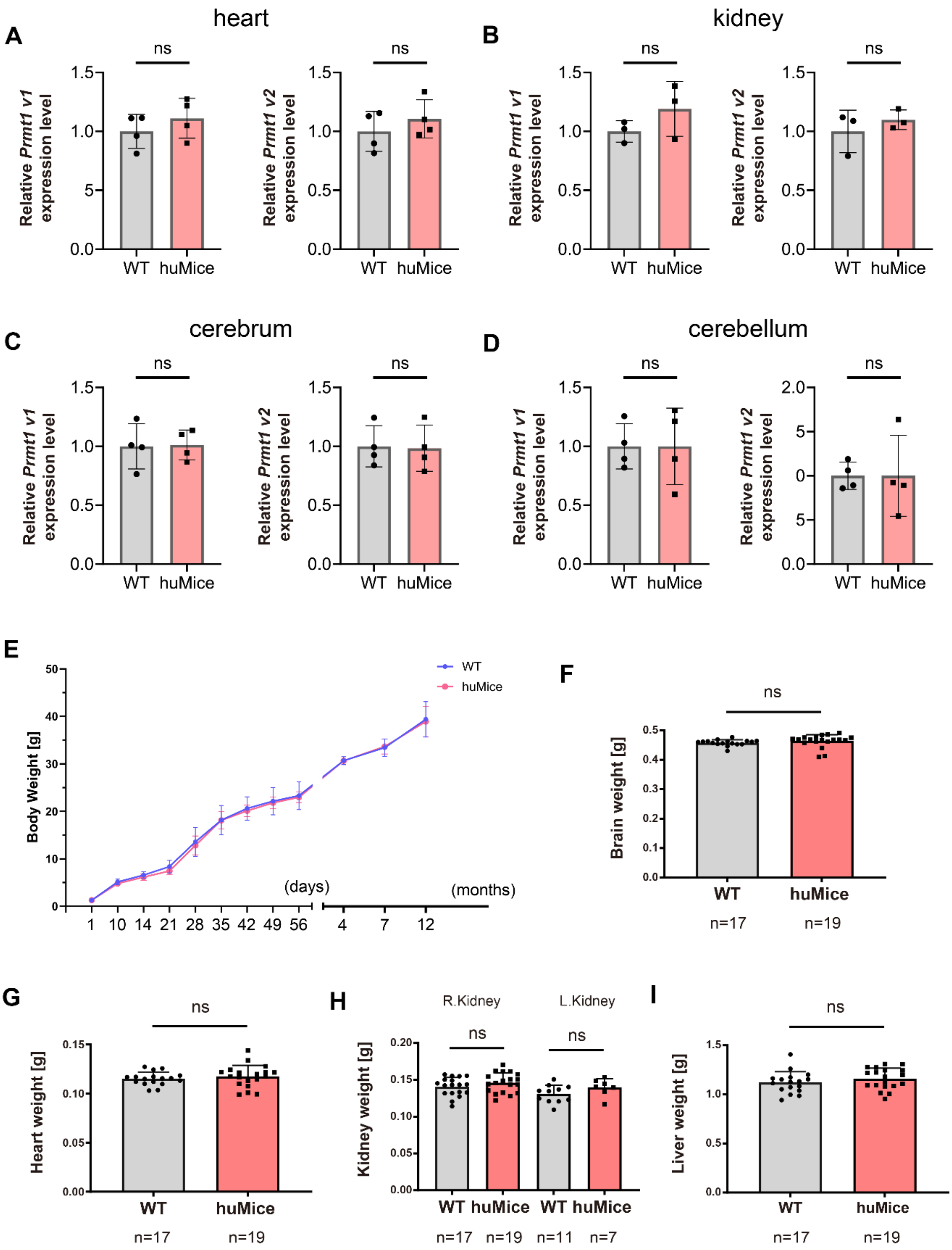

Figure S3. PRMT1 variant expression and organ development parameters in young huMice.

(A) mRNA expression levels of Prmt1 variant 1 and variant 2 in the hearts of male WT and huMice at 2 months of age (*n* =4), as assessed by quantitative real-time PCR (qRT-PCR). (B) mRNA expression levels of Prmt1 variant 1 and variant 2 in the kidneys of male WT and huMice at 2 months of age (*n* =3), as assessed by qRT-PCR. (C) mRNA expression levels of Prmt1 variant 1 and variant 2 in the cerebrums of male WT

and huMice at 2 months of age, as assessed by qRT-PCR (*n* =4). (D) mRNA expression levels of Prmt1 variant 1 and variant 2 in the cerebellums of male WT and huMice at 2 months of age, as assessed by qRT-PCR (*n* =4). (E) Body weight changes from postnatal day 1 to 1 year of age in male WT and huMice. (F) Weight of the brains in 2-month-old male WT and huMice. (G) Weight of the hearts in 2-month-old male WT and huMice. (H) Weight of the kidneys in 2-month-old male WT and huMice. (I) Weight of the livers in 2-month-old male WT and huMice.

All data are presented as mean ± SEM. Statistical analysis was performed using unpaired two-tailed *t*-test. *ns, no significant differences.*

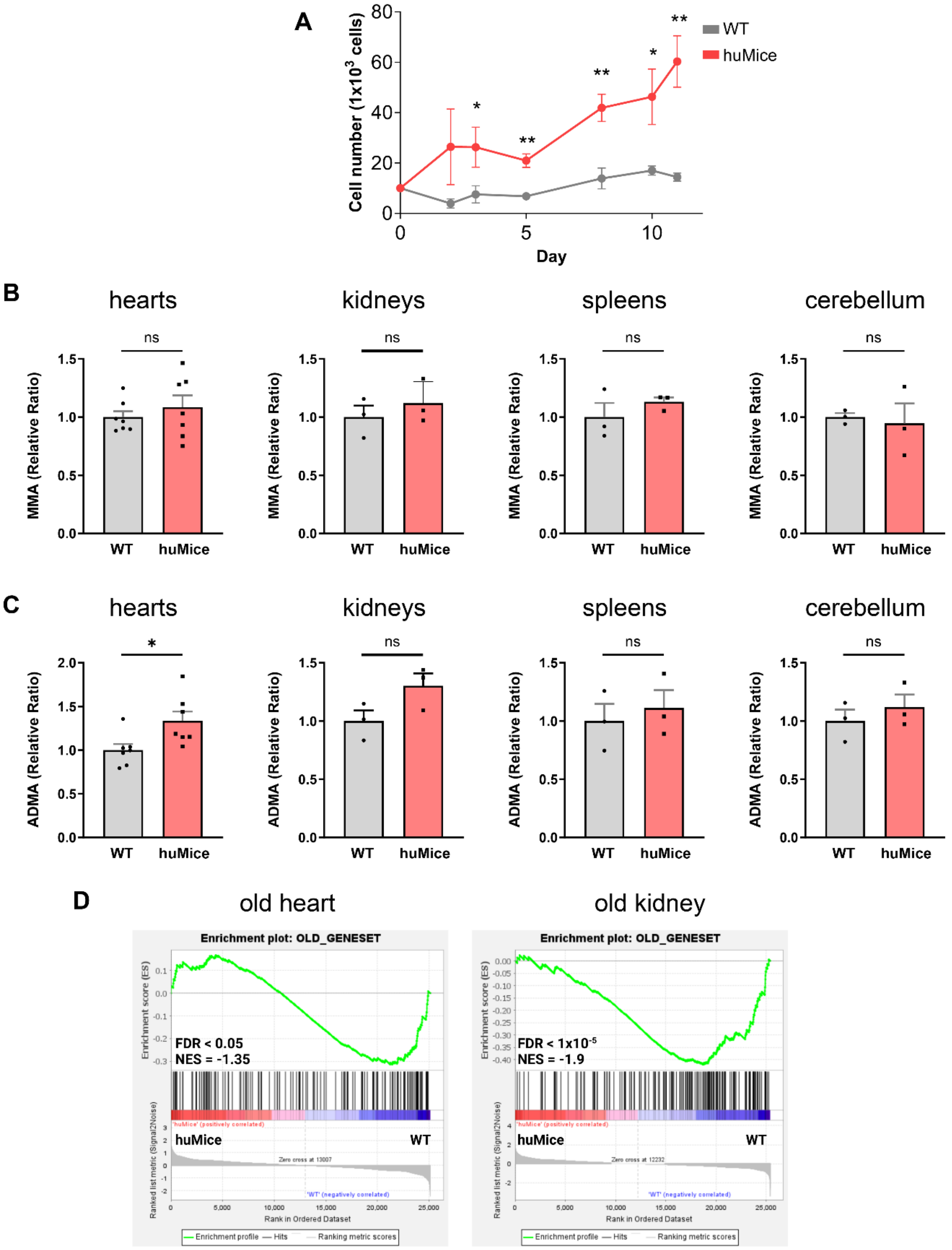

Figure S4. Cellular growth, arginine methylation levels, and transcriptomic aging signature in huMice.

(A) Cell growth curve of late-passage (passage 8) male MEFs derived from WT mice and huMice embryos at E12.5. $^{*}P < 0.05$, $^{**}P < 0.01$ by unpaired two-tailed *t*-test. Data represent mean ± SEM. (B) Quantification of monomethylarginine (MMA) levels in the hearts (n =7), kidneys, spleens, and cerebellums (n =3) of 2-month-old male WT and huMice using LC-MS/MS. Data represent mean ± SEM. No significant differences were

observed between the groups (*ns*, unpaired *t*-test). (C) Quantification of asymmetric dimethylarginine (ADMA) levels in the hearts (*n* =7), kidneys, spleens, and cerebellums (*n* =3) of 2-month-old male WT and huMice using LC-MS/MS. ADMA levels were significantly elevated in the hearts of huMice compared to WT mice (*$P < 0.05$, unpaired *t*-test), while no significant differences were detected in other organs (*ns*). Data are presented as mean ± SEM. (D) Gene Set Enrichment Analysis (GSEA) plots illustrating that the “age-associated gene set is downregulated in the heart and kidney of old (21-22 months) male huMice, compared to male WT mice. NES, normalized enrichment score.

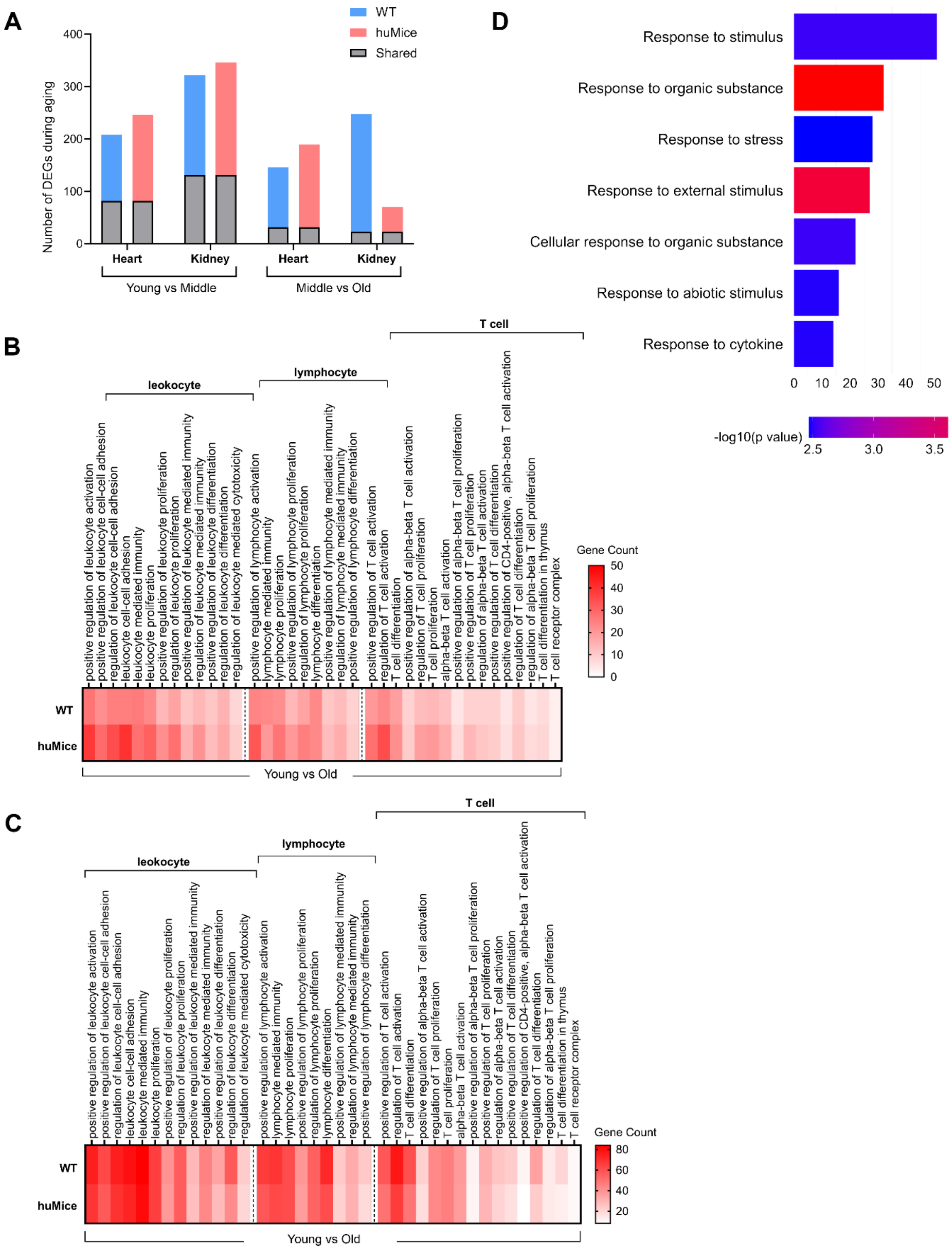

Figure S5. Age-dependent transcriptomic divergence between WT and huMice highlights immune regulatory pathways.

(A) Number of DEGs during aging in male WT and huMice. Gray sections represent DEGs shared between WT and huMice, blue sections represent DEGs unique to WT mice, and red sections represent DEGs unique to huMice. The left section shows changes from young (2 months) to middle-aged (12 months), while the right section

shows changes from middle-aged (12 months) to old (21–22 months). (B) Heatmap showing the enrichment of GO terms related to T-cell-, lymphocyte-, and leukocyte-mediated immunity in the hearts, comparing young (2 months) and old (21-22 months) mice. The color gradient indicates the number of genes contributing to each GO term. (C) Heatmap showing the enrichment of gene ontology (GO) terms related to T-cell-, lymphocyte-, and leukocyte-mediated immunity in the kidneys, comparing young (2 months) and old (21-22 months) mice. (D) Gene ontology (GO) analysis of 12-month-old hearts from male huMice and WT mice, highlighting significant differences in pathways related to inflammatory regulation.

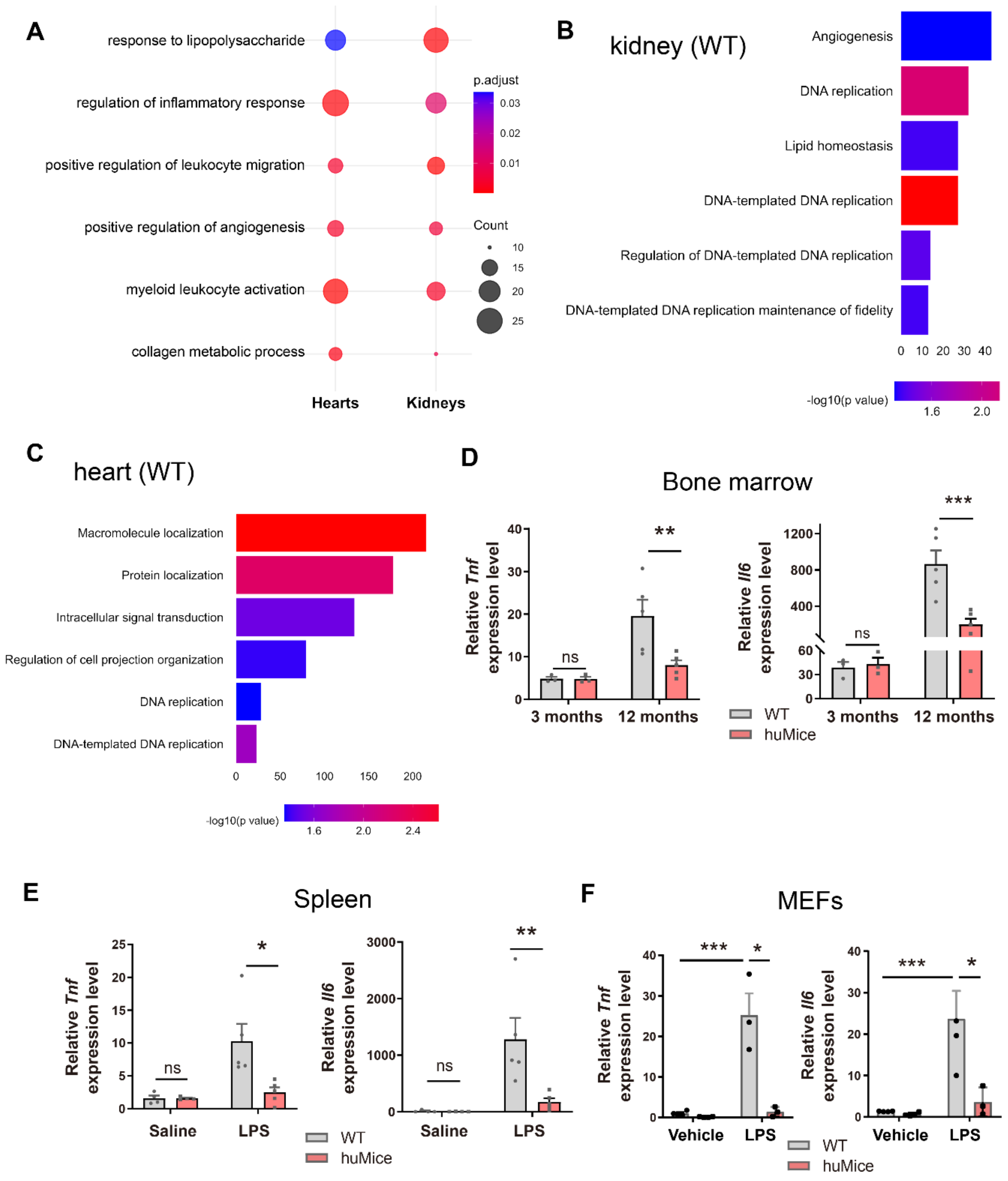

Figure S6. Divergent inflammatory gene expression responses to LPS challenge in WT and huMice.

(A) GO analysis of DEGs between LPS-treated male WT and huMice (12 months), showing inflammation-related functions like "Response to lipopolysaccharide", and "regulation of inflammatory response" commonly enriched in both heart and kidney tissues. (B,C) Gene Ontology (GO) enrichment analysis of WT-specific unique differentially expressed genes (DEGs) identified in the Venn diagram (Figure 6C). Both

in the heart (B) and kidney (C), these WT-specific DEGs are significantly enriched in pathways related to DNA replication, underscoring a distinct transcriptional response to inflammatory stimuli in WT mice (12 months). (D) mRNA expression levels of *Tnf* and *Il6* in the bone marrow of male WT and huMice 4 hours after LPS injection for both the 3-month-old and 12-month-old age groups, measured by qRT-PCR (3 months, n = 4; 12months, n =5). (E) mRNA expression levels of *Tnf* and *Il6* in the Spleen of male WT and huMice 4 hours after LPS injection for 12-month-old age group (Saline, n = 4; LPS, n =5), measured by qRT-PCR. (F) *Tnf* and *Il6* mRNA levels in male WT and huMice MEFs treated with 0.5 μg/ml LPS-EB for 24 h (n = 3-4).

All data are presented as mean ± SEM. Statistical analysis was performed using two-way ANOVA followed by Tukey's multiple comparisons test. *ns*, not significant. *$P < 0.05$, **$P < 0.01$, ***$P < 0.001$.

Enzyme	Product	Vmax (nM·min^{-1})	Km (μM)	kcat (×10^{-2}· min^{-1})	kcat/Km (×10^{-2} ·min^{-1}·μM)
mPRMT1	MMA	29.56	7.93	14.78	1.86
hPRMT1	MMA	31.66	8.26	15.82	1.92
mPRMT1	ADMA	1.23	1.93	0.61	0.32
hPRMT1	ADMA	1.54	1.6	0.77	0.48

Supplementary Table 1 (related to Figure S1). Steady-state kinetic parameters of human and mouse PRMT1 using the EWS-RGG peptide substrate.
